# Association of Immunosuppression and Viral Load With Subcortical Brain Volume in an International Sample of People Living With HIV

**DOI:** 10.1001/jamanetworkopen.2020.31190

**Published:** 2021-01-15

**Authors:** Talia M. Nir, Jean-Paul Fouche, Jintanat Ananworanich, Beau M. Ances, Jasmina Boban, Bruce J. Brew, Joga R. Chaganti, Linda Chang, Christopher R. K. Ching, Lucette A. Cysique, Thomas Ernst, Joshua Faskowitz, Vikash Gupta, Jaroslaw Harezlak, Jodi M. Heaps-Woodruff, Charles H. Hinkin, Jacqueline Hoare, John A. Joska, Kalpana J. Kallianpur, Taylor Kuhn, Hei Y. Lam, Meng Law, Christine Lebrun-Frénay, Andrew J. Levine, Lydiane Mondot, Beau K. Nakamoto, Bradford A. Navia, Xavier Pennec, Eric C. Porges, Lauren E. Salminen, Cecilia M. Shikuma, Wesley Surento, April D. Thames, Victor Valcour, Matteo Vassallo, Adam J. Woods, Paul M. Thompson, Ronald A. Cohen, Robert Paul, Dan J. Stein, Neda Jahanshad

**Affiliations:** 1 Imaging Genetics Center, Mark and Mary Stevens Neuroimaging and Informatics Institute, Keck School of Medicine, University of Southern California, Marina del Rey; 2 Department of Psychiatry and Neuroscience Institute, University of Cape Town, Cape Town, South Africa; 3 The Henry M. Jackson Foundation for the Advancement of Military Medicine, Bethesda, Maryland; 4 South East Asian Research Collaboration in HIV, Thai Red Cross AIDS Research Centre, Bangkok, Thailand; 5 AIGHD, University of Amsterdam, Amsterdam, the Netherlands; 6 Department of Neurology, Washington University School of Medicine, St Louis, Missouri; 7 Faculty of Medicine, Department of Radiology, University of Novi Sad, Novi Sad, Serbia; 8 Department of Neurology, St Vincent’s Hospital, St Vincent’s Health Australia and University of New South Wales, Sydney, New South Wales, Australia; 9 Department of Immunology, St Vincent’s Hospital, St Vincent’s Health Australia and University of New South Wales, Sydney, New South Wales, Australia; 10 Peter Duncan Neurosciences Unit, St Vincent’s Centre for Applied Medical Research, Sydney, New South Wales, Australia; 11 Department of Medical Imaging, St Vincent’s Hospital, University of New South Wales, Sydney, New South Wales, Australia; 12 Department of Diagnostic Radiology & Nuclear Medicine, University of Maryland School of Medicine, Baltimore; 13 Department of Neurology, University of Maryland School of Medicine, Baltimore; 14 Department of Medicine, John A. Burns School of Medicine, University of Hawaii, Manoa, Honolulu; 15 Department of Neurology, Johns Hopkins University School of Medicine, Baltimore, Maryland; 16 School of Psychology, University of New South Wales, Sydney, New South Wales, Australia; 17 Department of Epidemiology and Biostatistics, Indiana University School of Public Health, Bloomington; 18 Missouri Institute of Mental Health, University of Missouri, St Louis; 19 Department of Psychiatry and Biobehavioral Sciences, University of California, Los Angeles; 20 HIV Mental Health Research Unit, Department of Psychiatry and Mental Health, University of Cape Town, Cape Town, South Africa; 21 Hawaii Center for AIDS, University of Hawaii, Honolulu; 22 Department of Tropical Medicine, Medical Microbiology and Pharmacology, University of Hawaii, Honolulu; 23 Department of Radiology, Alfred Health, Monash University, Melbourne, Victoria, Australia; 24 Neurology, UR2CA, Centre Hospitalier Universitaire Pasteur 2, Université Nice Côte d’Azur, Nice, France; 25 Department of Neurology, University of California, Los Angeles; 26 Department of Radiology, UR2CA, Centre Hospitalier Universitaire Pasteur 2, Université Nice Côte d’Azur, Nice, France; 27 Infection Unit, School of Public Health, Tufts University Medical School, Boston, Massachusetts; 28 Cote d’Azur University, Sophia Antipolis, France; 29 Epione Team, Inria, Sophia Antipolis Mediterrannee, Sophia Antipolis, France; 30 Center for Cognitive Aging and Memory, Department of Clinical and Health Psychology, McKnight Brain Institute, University of Florida, Gainesville; 31 Department of Psychology, University of Southern California, Los Angeles; 32 Memory and Aging Center, Department of Neurology, University of California, San Francisco; 33 Global Brain Health Institute, San Francisco, California; 34 Internal Medicine/Infectious Diseases, Centre Hospitalier de Cannes, Cannes, France; 35 Psychological Sciences, Missouri Institute of Mental Health, University of Missouri, St Louis; 36 SA MRC Unit on Risk & Resilience in Mental Disorders, Department of Psychiatry & Neuroscience Institute, University of Cape Town, Cape Town, South Africa

## Abstract

**Importance:**

Despite more widely accessible combination antiretroviral therapy (cART), HIV-1 infection remains a global public health challenge. Even in treated patients with chronic HIV infection, neurocognitive impairment often persists, affecting quality of life. Identifying the neuroanatomical pathways associated with infection in vivo may delineate the neuropathologic processes underlying these deficits. However, published neuroimaging findings from relatively small, heterogeneous cohorts are inconsistent, limiting the generalizability of the conclusions drawn to date.

**Objective:**

To examine structural brain associations with the most commonly collected clinical assessments of HIV burden (CD4^+^ T-cell count and viral load), which are generalizable across demographically and clinically diverse HIV-infected individuals worldwide.

**Design, Setting, and Participants:**

This cross-sectional study established the HIV Working Group within the Enhancing Neuro Imaging Genetics Through Meta Analysis (ENIGMA) consortium to pool and harmonize data from existing HIV neuroimaging studies. In total, data from 1295 HIV-positive adults were contributed from 13 studies across Africa, Asia, Australia, Europe, and North America. Regional and whole brain segmentations were extracted from data sets as contributing studies joined the consortium on a rolling basis from November 1, 2014, to December 31, 2019.

**Main Outcomes and Measures:**

Volume estimates for 8 subcortical brain regions were extracted from T1-weighted magnetic resonance images to identify associations with blood plasma markers of current immunosuppression (CD4^+^ T-cell counts) or detectable plasma viral load (dVL) in HIV-positive participants. Post hoc sensitivity analyses stratified data by cART status.

**Results:**

After quality assurance, data from 1203 HIV-positive individuals (mean [SD] age, 45.7 [11.5] years; 880 [73.2%] male; 897 [74.6%] taking cART) remained. Lower current CD4^+^ cell counts were associated with smaller hippocampal (mean [SE] β = 16.66 [4.72] mm^3^ per 100 cells/mm^3^; *P* < .001) and thalamic (mean [SE] β = 32.24 [8.96] mm^3^ per 100 cells/mm^3^; *P* < .001) volumes and larger ventricles (mean [SE] β = −391.50 [122.58] mm^3^ per 100 cells/mm^3^; *P* = .001); in participants not taking cART, however, lower current CD4^+^ cell counts were associated with smaller putamen volumes (mean [SE] β = 57.34 [18.78] mm^3^ per 100 cells/mm^3^; *P* = .003). A dVL was associated with smaller hippocampal volumes (*d* = −0.17; *P* = .005); in participants taking cART, dVL was also associated with smaller amygdala volumes (*d* = −0.23; *P* = .004).

**Conclusions and Relevance:**

In a large-scale international population of HIV-positive individuals, volumes of structures in the limbic system were consistently associated with current plasma markers. Our findings extend beyond the classically implicated regions of the basal ganglia and may represent a generalizable brain signature of HIV infection in the cART era.

## Key Points

**Question:**

Are HIV plasma markers that are universally used to monitor immune function and treatment response associated with subcortical brain volumes in clinically and demographically heterogeneous HIV-infected individuals?

**Findings:**

In this cross-sectional study of 1203 HIV-infected adults, lower current CD4^+^ T-cell counts were associated with smaller hippocampal and thalamic volumes independent of treatment status, although in the subset of participants not receiving treatment, they were associated with smaller putamen volumes. Across all participants, detectable viral load was associated with smaller hippocampal volumes, but in the subset of participants receiving HIV treatment, detectable viral load was also associated with smaller amygdala volumes.

**Meaning:**

In a heterogeneous population of HIV-infected individuals, volumes of structures in the limbic system were consistently associated with plasma markers.

## Teaser

This cross-sectional study examines structural brain associations with CD4^+^ T-cell count and viral load in HIV-positive individuals taking and not taking combination antiretroviral therapy.

## Introduction

In the era of globally accessible combination antiretroviral therapy (cART), morbidity and mortality rates have markedly decreased for individuals who have contracted HIV-1. HIV-infected individuals taking cART are now expected to reach near-normal life expectancy.^1^ However, HIV-related comorbidities, including symptoms of brain dysfunction, remain common even in treated HIV-positive individuals.^2,3,4^

The prevalence of neurocognitive impairment in HIV-positive populations varies across studies: in a previous review,^5^ some reports suggested that cognitive difficulties are present in nearly half of HIV-positive individuals, but other studies reported that fewer than 20% are affected. This variability may reflect heterogeneous virus-host dynamics and cART treatment status. Other factors may include cultural, socioeconomic, and educational variability across clinical settings and geographic regions^5^ as well as neuropsychological testing methods, including the use of appropriate normative data, and the handling of neurologic and psychiatric confounders. Another review^3^ of studies that used standard neuropsychological assessments and appropriate normative data found robust associations between even mild neurocognitive impairment and interference with the capacity to perform instrumental activities of daily living.

HIV preferentially infects CD4^+^ T lymphocytes and damages the immune system. CD4^+^ T-cell count, a marker of immune function, and HIV viral load (VL) are 2 blood plasma biomarkers routinely collected from patients with HIV to monitor infection and treatment response. These clinical markers are also the most consistently available in human studies of HIV, but the degree to which they capture central nervous system (CNS) injury is not fully understood. Low nadir CD4^+^ cell counts have been identified as a factor associated with neurocognitive impairment in the cART era,^6^ suggesting that severe immunosuppression may lead to persistent and potentially irreversible brain injury. However, nadir CD4^+^ cell counts are frequently self-reported, so they may be unreliable or unknown. The duration of immunosuppression before recovery and the highest VL may also not be well documented. Immune restoration and viral suppression are tracked through routine clinical assessments and may be achieved through modifiable factors, including timely HIV testing and treatment initiation and adherence. The neurologic implications of maintaining or achieving healthy targets are therefore important to establish.

Structural neuroimaging provides a promising array of quantitative biomarkers for assessing CNS function and decline. Neuroimaging biomarkers have provided important noninvasive means to understand many psychiatric and neurodegenerative diseases and to monitor the effectiveness of clinical interventions.^7^ Neuroimaging studies of HIV-positive individuals typically find smaller subcortical volumes in infected individuals,^8^ in particular, in structures of the basal ganglia,^9,10,11^ and associations between such structural differences and neurocognitive impairment.^12^ However, in studies conducted during the cART era, inconsistencies in the effect sizes and regional distribution of brain abnormalities associated with CD4^+^ cell count or VL in HIV-positive individuals have limited the generalizability of the conclusions drawn to date. These inconsistent findings are likely driven in part by differences in demographic characteristics, such as age and sex, socioeconomic status, lifestyle factors, history of trauma, substance use, and clinical heterogeneity of study participants, including viral suppression, cART timing and adherence, CD4^+^ cell count range, and other comorbidities. For instance, a study^13^ of untreated, chronically infected individuals reported a positive association between thalamic volumes and current CD4^+^ T-cell counts, whereas another study^14^ evaluating a diverse sample reported negative associations with total subcortical volume (including the thalamus). However, the effects are not necessarily population specific because studies focusing on individuals receiving long-term treatment^15^ and those who are cART naïve^16^ have both reported no associations between CD4^+^ cell count and individual subcortical volumes. Different findings across studies may also reflect methodologic heterogeneity, including differences in (1) statistical power with variable sample sizes, in addition to unequal representation of important HIV disease factors and neurocognitive impairment prevalence; (2) magnetic resonance imaging (MRI) scanners and image acquisition protocols; and (3) image processing or statistical analysis techniques. By reducing methodologic variability in image processing, boosting sample size, and assessing a diverse set of HIV-positive cohorts, a generalizable pattern of HIV-related brain effects may be identified.

The HIV Working Group was established within the Enhancing Neuro Imaging Genetics through Meta Analysis (ENIGMA) consortium to pool data from neuroimaging studies using harmonized data analysis pipelines. The working group is a growing international collaboration open to all researchers investigating the neurologic consequences of HIV infection. This study included active involvement from investigators of 13 HIV studies across 6 different countries: the US, France, Serbia, Australia, Thailand, and South Africa (Table 1).^17,18,19,20,21,22^ We aimed to investigate structural brain volume associations with the most commonly collected clinical assessments of HIV burden; we surveyed plasma CD4^+^ T-cell counts and the detectability of viral RNA and assessed their associations with MRI-derived subcortical brain volumes in HIV-positive individuals. The strength of this large data set is that it is more representative of many HIV-positive individuals currently living in the cART era (including those who are not treated, with and without viral suppression) than any single cohort study. Subcortical regions remain a target of HIV infection; therefore, these structures represent an ideal benchmark against which the degree of immune recovery, viral suppression, and cART use may be assessed.

**Table 1.** Demographic and Clinical Information by Study and Scanning Site

Site	Total, No.	Male, No. (%)	Age, mean (SD) [range], y	No. (%) of participants		CD4^+^ cell count, mean (SD), /μL	No. (%) of participants	
				Taking cART	Detectable viral load		CD4^+^ cell count <200/μL	Viral load >400 copies/mL
HIVNC (7 sites), US	218	187 (86)	48.5 (8.3) [24-71]	218 (100)	61 (28) [n = 217]	378.0 (231.9)	48 (22)	39 (18) [n = 217]
Site 1: University of California, San Diego	21	21 (100)	47.6 (5.0) [37-56]	21 (100)	13 (65) [n = 20]	313.7 (328.4)	11 (52)	7 (35) [n = 20]
Site 2: Harbor UCLA Medical Center, Los Angeles, California	52	42 (81)	46.6 (9.1) [24-70]	52 (100)	8 (15)	350.2 (173.0)	14 (27)	5 (10)
Site 3: Stanford University, Stanford, California	10	9 (90)	46.4 (10.4) [31-62]	10 (100)	0	313.5 (157.5)	2 (20)	0
Site 4: Colorado	35	34 (97)	49.2 (7.9) [31-66]	35 (100)	14 (40)	446.6 (246.0)	4 (11)	6 (17)
Site 5: Pittsburgh, Pennsylvania	19	18 (95)	49.9 (9.7) [34-71]	19 (100)	4 (21)	438.9 (298.2)	3 (16)	3 (16)
Site 6: Rochester University, Rochester, New York	41	26 (63)	48.4 (7.7) [26-62]	41 (100)	11 (27)	382.0 (207.8)	6 (15)	7 (17)
Site 7: University of California, Los Angeles	40	37 (93)	50.7 (8.4) [28-69]	40 (100)	11 (28)	370.7 (224.7)	8 (20)	11 (28)
University of Hawaii, Honolulu, US^17^	175	159 (91)	47.6 (10.5) [20-74]	157 (90)	54 (33) [n = 164]	475.2 (281.4)	31 (18)	38 (23) [n = 164]
University of Hawaii, Honolulu, US^18^	53	45 (85)	50.9 (8.0) [40-71]	53 (100)	6 (11)	491.1 (208.5)	4 (8)	2 (4)
University of California, San Francisco, US	50	49 (98)	63.6 (2.5) [60-69]	49 (98)	14 (29) [n = 49]	529.3 (218.5)	0	4 (8) [n = 49]
Brown University, Providence, Rhode Island, US	79	48 (61)	45.2 (9.5) [23-65]	66 (84)	22 (28)	476.2 (228.8)	7 (9)	21 (27)
University of California, Los Angeles, US^19^	12	12 (100)	46.2 (8.5) [26-57]	9 (75)	6 (50)	604.1 (289.1)	1 (8)	4 (33)
University of California, Los Angeles, US^20^	51	46 (90)	50.9 (13.2) [24-76]	51 (100)	21 (41)	603.2 (287.7)	2 (4)	9 (18)
University of New South Wales, New South Wales, Australia^21^	39	38 (97)	52.8 (8.2) [39-75]	39 (100)	10 (26)	612.5 (269.0)	0	6 (15)
University of New South Wales, New South Wales, Australia^22^	68	68 (100)	55.3 (6.7) [44-69]	68 (100)	1 (1)	549.5 (273.6)	5 (7)	1 (1)
SEARCH-011 Consortium, Bangkok, Thailand	61	26 (43)	34.2 (7.0) [22-56]	0	61 (100)	236.0 (139.0)	25 (41)	61 (100)
University of Cape Town, South Africa	181	25 (14)	32.4 (5.0) [22-46]	0	148 (97) [n = 152]	225.5 (149.2)	95 (53)	129 (88) [n = 147]
Nice University, Nice, France	155	122 (79)	45.4 (10.0) [22-81]	126 (76)	56 (36)	580.9 (277.6)	11 (7)	39 (25)
University of Novi Sad, Novi Sad, Serbia	61	55 (90)	44.3 (10.9) [25-66]	61 (100)	10 (16)	616.3 (346.2)	1 (2)	10 (16)

Abbreviations: cART, combination antiretroviral therapy; HIVNC, HIV Neuroimaging Consortium; SEARCH, South East Asian Research Collaboration in HIV.

## Methods

### Participants and Clinical Assessments

In this cross-sectional study, T1-weighted brain MRI scans and clinical data from 1295 HIV-positive adult participants were collected at 19 sites from 13 independent studies. The participating studies included the multisite HIV Neuroimaging Consortium (7 sites), Brown University, University of California, San Francisco, 2 groups from the University of Hawaii, and 2 groups from the University of California, Los Angeles, all in the US; Nice University Hospital in France; the University of Cape Town in South Africa; 2 groups from the University of South Wales in Australia; University of Novi Sad in Serbia; and the South East Asian Research Collaboration in HIV 011 study from Thailand. Inclusion and exclusion criteria for each study are summarized in eTable 1 in the Supplement. Clinical assessments at the time of MRI included current CD4^+^ T-cell counts and HIV plasma RNA VL. Each study’s researchers obtained approval from their local ethics committee or institutional review board; participants signed an informed consent form at each participating site. All data were deidentified. This study followed the Strengthening the Reporting of Observational Studies in Epidemiology (STROBE) reporting guideline.

### Image Acquisition, Processing, and Quality Assurance

T1-weighted brain images were acquired at each site on a 3-T or 1.5-T scanner. Acquisition protocols are detailed in eTable 2 in the Supplement. Intracranial volume was estimated and regional segmentations performed using FreeSurfer software, version 5.3^23^ on a rolling basis from November 1, 2014, to December 31, 2019, as contributing studies joined the consortium. Volumes were extracted from 8 regions of interest: thalamus, caudate, putamen, globus pallidus, hippocampus, amygdala, nucleus accumbens, and lateral ventricles. Because no lateralized effects were hypothesized, the mean of the left and right volumes for each region of interest was evaluated. Volume extraction and quality control were completed by each respective site using the publicly available protocol under the heading ENIGMA Subcortical Segmentation Protocols for Disease Working Groups (http://enigma.ini.usc.edu/protocols/imaging-protocols). Participants excluded because of missing or poor quality data are summarized in eTable 3 in the Supplement. Quality assurance also included a feasibility study to ensure that the pooled sample was able to capture known negative associations between brain volume and age (eMethods and eResults in the Supplement).

### Statistical Analysis

#### Associations Between Brain Volumes and HIV Plasma Markers

Random-effects multivariable linear regressions were performed to evaluate associations between each of the 8 regional brain volumes and (1) current CD4^+^ T-cell count or (2) a binary variable indicating a detectable or undetectable VL (1 or 0, respectively). For each participant, VL was determined to be detectable according to the detection threshold at the respective collection site, which varied (range, 19-400 copies/mL) (eTable 4 in the Supplement). To account for differences in MRI scanner and acquisition, MRI collection site was used as the random-effects grouping variable; fixed-effects covariates included age, sex, the interaction between age and sex, cART status at the time of scan (taking or not taking cART), and estimated total intracranial volume to adjust for variability in head size. Statistical analyses were conducted with the nlme package in R, version 3.2.3 (R Foundation for Statistical Computing). Effect sizes, after accounting for all covariates, were estimated as *d* values for dichotomous factors and *r* values (partial correlation coefficients) for continuous variables (eMethods and eResults in the Supplement). Bonferroni correction was used to control the family-wise error rate at 5%; an uncorrected, 2-sided *P* ≤ .0063 (.05 divided by 8; ie, the number of brain regions) was considered statistically significant.

#### Post Hoc Sensitivity Analyses: Stratification by cART Status and Sex

Post hoc analyses tested for associations between brain volumes and HIV plasma markers when the data were stratified by (1) cART status and (2) sex. The same analytical framework was used, removing sex and sex × age interactions from statistical models when stratifying by sex and removing cART when stratifying by cART status. Group differences in demographic and clinical factors were evaluated with χ^2^ tests for dichotomous factors and 2-tailed *t* tests for continuous measures.

#### Validation Analyses Comparing Harmonized Thresholds

Two validation analyses were performed: (1) dichotomizing CD4^+^ cell count based on the AIDS-defining threshold of 200/μL and (2) defining a common detectable VL (dVL) threshold across sites (400 copies/mL, the highest site-specific assay detection threshold used across all collection sites).

## Results

Demographic and clinical characteristics for participants included from each of the 13 studies after quality assurance are reported in Table 1 and summarized for the entire ENIGMA-HIV cohort (N = 1203; mean [SD] age, 45.7 [11.5] years; 880 [73.2%] male; 897 [74.6%] taking cART) in Table 2. Compared with the 897 participants taking cART and the 880 male participants, 306 participants not taking cART and 323 female participants were younger, had lower CD4^+^ cell counts, and were more likely to have a detectable VL. Proportionally fewer female participants were taking cART at the time of MRI. As expected, individuals with a dVL had significantly lower CD4^+^ cell counts than those with an undetectable VL (mean [SE] CD4^+^ cell count in dVL, 336.3/μL [221.5/μL]; mean [SE] CD4^+^ cell count in undetectable VL, 532.3/μL [283.1/μL]; *P* < .001). In addition, as expected, across participants, older age was associated with smaller volumes of all subcortical structures and larger ventricular volumes. Results from the age feasibility analysis are presented in eTable 5 and eFigures 1 and 2 in the Supplement. Although we did not have HIV-negative controls, we used the lifespan centile curves reported in a large, multisite study from the ENIGMA lifespan working group^24^ and determined that subcortical volumes of our HIV-positive population did not fall within the same distribution as those from age-matched healthy individuals (eFigures 3, 4, 5, and 6 and eTable 6 in the Supplement).

**Table 2.** Summary of Demographic, Clinical, and Neuroanatomical Information Aggregated Across All 13 Participating Studies of HIV-Positive Adults and Stratified by cART Status and Sex^a^

Variable	cART status			*P* value for taking vs not taking cART^b^	Sex		*P* value for male vs female^b^
	Total (N = 1203)	Taking cART (n = 897)	Not taking cART (n = 306)		Male (n = 880)	Female (n = 323)	
Age, mean (SD) [range], y	45.7 (11.5) [20-81]	49.5 (10.0) [22-81]	34.7 (8.0) [20-66]	<.001	48.3 (10.8) [20-81]	38.7 (10.6) [22-70]	<.001
No. (%) taking cART	897 (74.6)	897 (100)	NA	NA	774 (88.0)	123 (38.1)	<.001
No. (%) with detectable viral load	470 (40.5) [N = 1161]	201 (22.7) [N = 884]	269 (97.1) [N = 277]	<.001	275 (31.8) [N = 865]	195 (65.9) [N = 296]	<.001
No. (%) with viral load >400 copies/mL	363 (31.4) [N = 1156]	115 (13.0) [N = 884]	248 (91.1) [N = 272]	<.001	188 (21.7) [N = 863]	177 (59.7) [N = 293]	<.001
CD4^+^ cell count, mean (SD), /μL	447.0 (277.1)	498.9 (278.9)	295.5 (207.3)	<.001	480.2 (276.2)	356.7 (259.3)	<.001
No. (%) with CD4^+^ cell count <200/μL	230 (19.1)	107 (11.9)	123 (40.2)	<.001	131 (14.9)	99 (30.7)	<.001
Volume, mean (SD), mm^3^							
Thalamus	7121.3 (995.3)	7256.7 (986.9)	6724.7 (911.6)	<.001	7344.6 (971.8)	6513.1 (782.6)	<.001
Caudate	3595.4 (496.4)	3623.5 (498.6)	3513.1 (481.0)	<.001	3654.4 (495.5)	3434.8 (462.8)	<.001
Putamen	5199.1 (724.5)	5176.2 (754.9)	5266.2 (623.4)	.04	5254.9 (751.7)	5047.2 (620.7)	<.001
Globus pallidus	1541.0 (252.3)	1511.5 (252.2)	1627.6 (232.2)	<.001	1547.2 (255.4)	1524.3 (242.9)	.15
Hippocampus	4049.6 (546.7)	4147.7 (497.5)	3762.2 (582.6)	<.001	4191.1 (504.9)	3664.2 (464.8)	<.001
Amygdala	1549.8 (286.9)	1605.2 (286.2)	1387.5 (219.4)	<.001	1624.8 (274.6)	1345.4 (209.9)	<.001
Accumbens	536.8 (123.9)	524.1 (129.0)	574.2 (98.4)	<.001	534.7 (129.4)	542.7 (107.2)	.28
Lateral ventricles	19 554.4 (12 529.0)	21 331.1 (12 859.3)	14 346.3 (9816.1)	<.001	21 528.3 (12 987.9)	14 176.7 (9264.9)	<.001

Abbreviations: cART, combination antiretroviral therapy; NA, not applicable.

^a^ Subcortical volume comparisons in this table are absolute and were not corrected for estimated total intracranial volume, as was done in the formal analyses.

^b^ Differences in dichotomous factors were assessed using χ^2^ tests; for continuous measures, differences were assessed using 2-tailed *t* tests.

### Associations Between Brain Volumes and HIV Plasma Measures

Across all 1203 participants, lower CD4^+^ cell counts were associated with smaller hippocampal (*r =* 0.10; *P* < .001) (Figure, A and Table 3) and thalamic (*r =* 0.10; *P* < .001) volumes and larger ventricular volumes (*r =* −0.093; *P* = .001). A dVL (n = 1161) was associated with smaller hippocampal volumes (*d =* −0.17; *P* = .005) (Figure, B and Table 4). For individual site effects, see eFigures 7 and 8 in the Supplement. As hypothesized, post hoc analyses revealed no lateralized effects in significant regions (eTable 7 in the Supplement).

**Figure.**
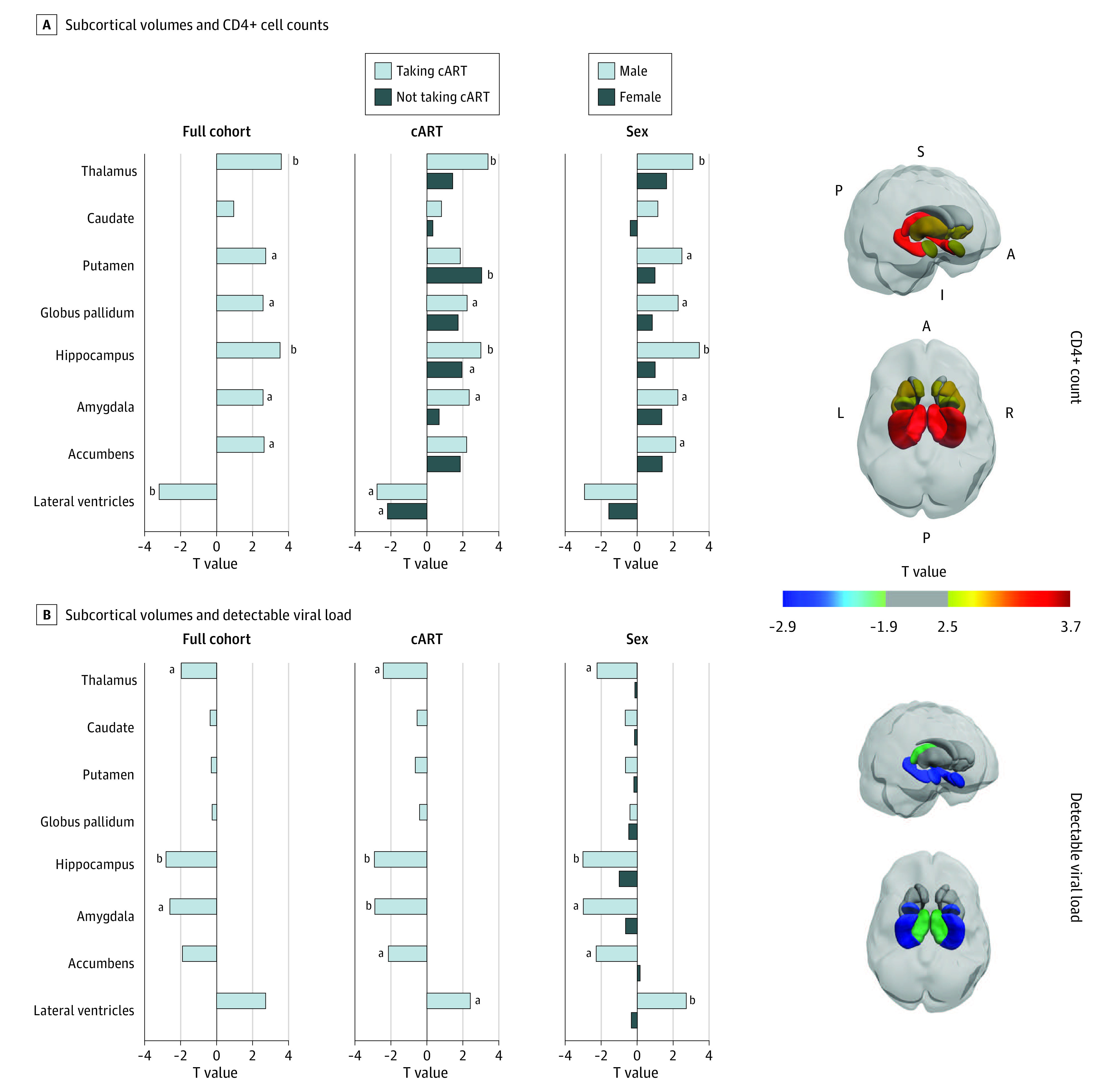
Associations Between Subcortical Volumes and CD4^+^ Cell Count and Detectable Viral Load at the Time of Scan T values are shown for all participants with HIV and separately in the subsets of participants taking combination antiretroviral therapy (cART), participants not taking cART, male participants, and female participants. We did not assess viral load in those not receiving treatment because of the limited number of individuals in this subgroup with undetectable viral load (n = 8). Three-dimensional brain visualizations show T values for subcortical structures that had associations at *P* ≤ .05 across all participants with HIV; those with association at *P* > .05 are depicted in gray. A indicates anterior; I, inferior; L, left; P, posterior; R, right; S, superior. ^a^*P* < .05. ^b^Significant at a Bonferroni corrected threshold for tests in 8 ROIs (*P* ≤ .0063).

**Table 3.** Partial Correlation Coefficients (*r*) and Unstandardized Regression Slopes Reflecting Change in Volume for Every 100/μL Change in CD4^+^ Cell Count (β) for Associations Between Regional Brain Volumes and CD4^+^ Cell Count at the Time of Magnetic Resonance Imaging Across All HIV-Positive Participants and by cART Status and Sex

ROI	Total (n = 1203)			Taking cART (n = 897)			Not taking cART (n = 306)			Male (n = 880)			Female (n = 323)		
	*r*	β (SE), mm^3^	*P* value	*r*	β (SE), mm^3^	*P* value	*r*	β (SE), mm^3^	*P* value	*r*	β (SE), mm^3^	*P* value	*r*	β (SE), mm^3^	*P* value
Thalamus	0.10	32.24 (8.96)	<.001^a^	0.11	30.08 (8.83)	<.001	0.084	39.4 (27.33)	.15	0.11	29.9 (9.66)	.002^a^	0.094	31.61 (19.31)	.10
Caudate	0.028	4.68 (4.92)	.34	0.028	4.14 (5.07)	.41	0.02	4.89 (14.56)	.74	0.039	6.2 (5.36)	.25	−0.022	−4.24 (10.96)	.70
Putamen	0.079	18.83 (6.89)	.0064	0.064	13.89 (7.36)	.06	0.18	57.34 (18.78)	.003^a^	0.085	19.57 (7.86)	.01	0.057	13.78 (13.9)	.32
Globus pallidus	0.075	6.44 (2.49)	.01	0.076	5.99 (2.67)	.02	0.10	12.03 (6.91)	.08	0.077	6.4 (2.82)	.02	0.048	4.33 (5.14)	.40
Hippocampus	0.10	16.66 (4.72)	<.001^a^	0.10	14.68 (4.88)	.003^a^	0.12	29.18 (14.34)	.04	0.12	18.15 (5.25)	<.001^a^	0.058	10.17 (10.02)	.31
Amygdala	0.075	5.63 (2.18)	.01	0.079	5.40 (2.29)	.02	0.04	4.26 (6.18)	.49	0.077	5.5 (2.43)	.02	0.078	6.29 (4.59)	.17
Accumbens	0.077	2.85 (1.08)	.008	0.075	2.53 (1.14)	.03	0.11	5.97 (3.15)	.06	0.073	2.66 (1.24)	.03	0.080	3.11 (2.23)	.16
Lateral ventricles	−0.093	−391.50 (122.58)	.001^a^	−0.094	−369.8 (132.25)	.005^a^	−0.13	−688.14 (314.35)	.03	−0.10	−417.5 (142.47)	.004^a^	−0.090	−356.96 (226.48)	.12

Abbreviations: cART, combination antiretroviral therapy; ROI, region of interest.

^a^ Significant at a Bonferroni corrected threshold for tests in 8 ROIs (*P* ≤ .0063).

**Table 4.** Effect Sizes (*d*) for Associations Between Regional Brain Volumes and Detectable Viral Load at the Time of Magnetic Resonance Imaging Across All HIV-Positive Participants and by cART Status and Sex^a^

ROI	Total (N = 1161)		Taking cART (n = 884)		Male (n = 865)		Female (n = 296)	
	*d* (SE)	*P* value	*d* (SE)	*P* value	*d* (SE)	*P* value	*d* (SE)	*P* value
Thalamus	−0.12 (0.060)	.046	−0.20 (0.080)	.02	−0.16 (0.073)	.03	−0.015 (0.12)	.90
Caudate	−0.022 (0.060)	.71	−0.044 (0.080)	.59	−0.049 (0.073)	.51	−0.018 (0.12)	.89
Putamen	−0.019 (0.060)	.76	−0.051 (0.080)	.53	−0.048 (0.073)	.52	−0.021 (0.12)	.87
Globus pallidus	−0.015 (0.060)	.80	−0.031 (0.080)	.70	−0.029 (0.073)	.70	−0.057 (0.12)	.66
Hippocampus	−0.17 (0.060)	.005^b^	−0.24 (0.080)	.004^b^	−0.22 (0.073)	.003^b^	−0.12 (0.12)	.33
Amygdala	−0.16 (0.060)	.009	−0.23 (0.080)	.004^b^	−0.22 (0.073)	.003^b^	−0.082 (0.12)	.52
Accumbens	−0.12 (0.060)	.06	−0.17 (0.080)	.03	−0.17 (0.073)	.02	0.022 (0.12)	.87
Lateral ventricles	0.16 (0.060)	.0065	0.20 (0.080)	.02	0.20 (0.073)	.0063^b^	−0.041 (0.12)	.75

Abbreviations: cART, combination antiretroviral therapy; ROI, region of interest.

^a^ We did not assess viral load in those not receiving treatment because of the limited number of individuals in this subgroup with undetectable viral load (n = 8).

^b^ Significant at a Bonferroni-corrected threshold for tests in 8 ROIs (*P* ≤ .0063).

### Post Hoc Sensitivity Analyses: Stratification by cART Status and Sex

Table 3 details regional CD4^+^ cell findings within stratified groups. As in the full group, in the subset of participants taking cART, lower CD4^+^ cell counts were associated with smaller hippocampal (*r =* 0.10; *P* = .003) and thalamic (*r =* 0.11; *P* < .001) volumes and larger ventricular volumes (*r =* −0.094; *P* = .005). However, in participants not taking cART, lower CD4^+^ cell counts were associated only with smaller putamen volumes (*r =* 0.18; *P* = .002).

Table 4 details regional dVL findings within the stratified groups. The hippocampal associations detected in the full group were also observed in participants taking cART (*d =* −0.24; *P* = .004), along with smaller amygdala volumes (*d =* −0.23; *P* = .004). Only 8 participants not taking cART had an undetectable VL; therefore, dVL in the group not taking cART was not assessed.

Because participants taking cART were largely male (n = 774 [88.0%]), associations in male participants mirrored those found in the subgroup taking cART: CD4^+^ cell counts were associated with hippocampal (*r =* 0.12; *P* < .001), thalamic (*r =* 0.11; *P* = .002), and ventricular (*r =* −0.10; *P* = .004) volumes; a dVL was associated with hippocampal (*d =* −0.22; *P* = .003) and amygdala (*d =* −0.22; *P* = .003) volumes. Ventricular volume effects (*d =* 0.20; *P* = .006) were significant only in the male subgroup but not the subgroup taking cART. No significant associations between plasma measures and brain volumes were detected in female participants, although a similar pattern in the ranking of regional effect sizes was observed between male and female participants for CD4^+^ cell count (Pearson *r =* 0.91; *P* = .002) (eFigure 9 in the Supplement).

### Validation Analyses

Validation analyses largely confirmed primary results. eTables 8 and 9 in the Supplement give the regional associations with dichotomized CD4^+^ cell counts (≤200/μL) and a harmonized dVL threshold (>400 copies/mL).

## Discussion

In this cross-sectional study, one of the largest coordinated brain imaging studies of HIV-positive individuals worldwide, data from 1203 individuals across 6 countries were harmonized and pooled to find associations between brain volumes and plasma markers used to monitor HIV infection. Brain volume associations with both CD4^+^ cell counts and dVL implicated the limbic system, despite the small effect sizes. Lower CD4^+^ cell counts were associated with smaller hippocampal and thalamic volumes in addition to larger ventricles, and a dVL was also associated with smaller hippocampal volumes. Sensitivity analyses stratifying the data by cART status revealed that the limbic associations were in large part attributable to the participants who were taking cART. Stratification also revealed diverging profiles for regions just shy of statistical significance in the full group. CD4^+^ cell count associations with putamen volumes were significant in the group not taking cART. Conversely, in the participants taking cART, dVL was associated with both hippocampal and amygdala volumes.

Plasma CD4^+^ T-cell count and VL are routinely assessed in HIV clinics around the world. These peripheral markers have been correlated with neuropsychological performance in HIV-positive individuals^2,25^ as well as postmortem brain tissue VL and pathology.^26,27^ Unfortunately, the association between plasma markers and MRI brain volume variation has been inconsistent across studies. Differences among studies, including clinical and demographic differences, methodologic variability, and/or insufficient power to estimate robust effect sizes, continue to complicate understanding of the neuroanatomical and neurofunctional consequences of HIV infection.

Brain signatures that generalize beyond individual studies are important to identify to establish neuroimaging biomarkers of HIV brain injury. Literature-based meta-analyses assess consistency of published findings and offer initial insights into the generalizability of HIV-related findings. A recent such meta-analysis^8^ of 19 published studies, spanning almost 3 decades, found significantly smaller total brain volumes and total gray matter volumes in HIV-positive individuals compared with seronegative controls; however, no significant serostatus associations with the basal ganglia (caudate, putamen, and globus pallidus) volumes were found. In addition, effect sizes were lower in more recent studies compared with earlier studies,^8^ which may suggest a diverging profile of HIV-affected neurocircuitry in more recent years.

The scope and extent of HIV-related neurocognitive impairment have evolved from the pre-cART era, and some individuals with chronic HIV in the cART era carry this legacy. For example, although the prevalence of neurocognitive deficits may be similar, reports^2,28^ have highlighted that learning deficits and poorer mental flexibility were more prominent features in patients treated with cART compared with patients in the pre-cART era in whom deficits in motor and sustained attention and psychomotor slowing were more evident. Brain deficits may persist despite cART because of factors such as possible cART neurotoxicity^5,29^; irreversible brain damage associated with advanced disease^6,8^; reservoirs of ongoing low-grade viral replication and/or persistent immune activation in the CNS^30,31^; vascular injury^32^; and neurodegenerative processes that can occur with aging,^33,34^ consistent with the hippocampal findings presented here.

In the pre-cART era, HIV-associated dementia was characterized as a subcortical dementia. In line with reported postmortem neuropathologic and viral protein distributions, neuroimaging studies of HIV-positive individuals often found smaller basal ganglia volumes,^9,10,35,36,37^ in particular smaller caudate nuclei.^11^ More recently, neuroimaging findings in HIV-positive individuals have extended beyond volume differences of the putamen, globus pallidus, nucleus accumbens, and caudate. HIV serostatus–related differences have been detected to varying degrees in the thalamus,^38^ amygdala,^39^ hippocampus,^40^ and even total gray matter,^8^ indicating that recent brain alterations may be more dispersed. These findings may also suggest greater heterogeneity in the causes of brain injury. Once a staple of reported HIV-related brain alterations, basal ganglia volume associations with CD4^+^ cell count or dVL did not survive correction for multiple comparisons in the full group of HIV-positive participants assessed here but remained significant in the subset not taking cART. Participants not taking cART were not necessarily cART naive, but their plasma markers may, on average, be more in line with those recorded before cART initiation. Although the CD4^+^ cell counts in this study were associated with altered limbic and not basal ganglia volumes in the full set of participants, it is possible that in these same individuals, nadir CD4^+^ cell counts, reflecting a history of severe immunosuppression before treatment, would be associated with basal ganglia volumes. The shift in HIV infection from fatal to chronic in the cART era appears to be accompanied by a shift in the profile of HIV-related brain abnormalities beyond the basal ganglia, frequently implicated in the pre-cART era, to limbic structures. This shift in subcortical signatures may be contributing to the increasing range of neuropsychiatric and cognitive outcomes.

In the current study, male study participants may have been more representative of infected individuals in the cART era: 88.0% of them were taking cART compared with only 38.1% of female participants, most of whom were recruited from Thailand and South Africa, countries in which a substantial proportion of individuals eligible for treatment may not receive it. This study found a similar pattern in the ranking of regional CD4^+^ cell count effect sizes between sexes but not dVL. As suggested in a US-based cohort of HIV-positive women,^41^ demographic and sociocultural factors, which are themselves associated with many comorbid conditions, potentially mask the effects of HIV disease. Despite being the largest neuroimaging evaluation of HIV-positive women, the current study did not detect any significant associations between plasma markers and brain volumes in female participants alone. This apparent difference in power may be attributable to any number of confounding factors. For example, comorbidities, including mental health conditions, may play a confounding role. Female-specific findings were noted in an international study^42^ in which women with posttraumatic stress disorder were found to be driving case-control differences in hippocampal volumes. Trauma, particularly that related to intimate partner violence, is overwhelmingly common among HIV-positive women,^43^ so factors such as comorbid posttraumatic stress disorder may be confounding limbic associations in women. Women constitute 52% of all individuals 15 years and older living with HIV.^44^ Nevertheless, women are underrepresented in HIV research, impeding the reliability and generalizability of findings. Despite evaluating more than 300 HIV-positive women, twice as many participants included in this study were men; only 2 of the 13 studies had recruited more than 40% women. A more extensive international effort assessing the neurologic effects of HIV infection in women is needed.

Effects in limbic structures, as seen here, have been detected in a wide range of clinical conditions studied in similar large-scale international efforts from the ENIGMA consortium.^45^ Serious mental illnesses, such as depression or substance use disorders, are highly prevalent in HIV-positive individuals and may increase the risk of HIV transmission.^46^ Because this study assessed individuals with and without these comorbidities, limbic associations detected in this study were not likely simply a reflection of such comorbid neuropsychiatric conditions. However, viral-induced immunosuppression may also contribute to the risk of developing serious mental illnesses by targeting the same neurocircuitry implicated across these disorders.

The hippocampus had consistent associations with both CD4^+^ cell count and VL measures. In postmortem studies, hippocampal tissue has some of the highest viral concentrations.^47^ Hippocampal neurons also have increased gliosis and HIV chemokine coreceptors and expression^48^ and may be particularly susceptible to Tat-induced apoptosis.^49^ A previous cART-era pathologic study^50^ suggests a potential shift in HIV-related inflammation to the hippocampus and surrounding entorhinal cortex. Hippocampal atrophy is consistently reported across aging populations, and accelerated atrophy is a hallmark of neurodegenerative diseases, such as Alzheimer disease. Neuropathologic hallmarks of healthy aging and Alzheimer disease, including elevated levels of phosphorylated tau and β-amyloid deposits, have been detected in the hippocampus of HIV-positive individuals taking cART.^51,52^ Common age- and HIV-related pathologic processes, such as inflammation and blood-brain barrier impairment, may accelerate age-related neurodegenerative processes.^34^ Study participants ranged in age from 20 to 81 years, and although age was associated with smaller brain volumes, the study did not detect any interactions between age and CD4^+^ cell count or dVL in the full group, perhaps because the subgroup taking cART was older than the group not taking cART. A better understanding of chronic infection in the context of aging remains an important topic of research.

### Limitations

Literature-based meta-analyses provide substantial insights into the reproducibility and consistency of published findings. However, they are inherently limited by the fact that effects are likely overestimated because of publication biases, given that studies with null findings are often excluded. Effect sizes reported in this study are considered small to moderate, yet given the large, diverse sample, small effect sizes may still be clinically significant. Furthermore, methodologic factors, including image analysis methods and statistical design, cannot be harmonized in retrospective analyses. In this study, these limitations were partially addressed by harmonizing image processing and statistical analyses. Nonetheless, several limitations and challenges remain. The current study focused only on associations in HIV-positive individuals, without a direct comparison to seronegative controls. Serostatus comparisons may help elucidate the full extent of HIV-related brain deficits, as opposed to highlighting regions more selectively affected by the degree of immunosuppression and viral control. Most of the HIV cohorts included in ENIGMA-HIV at the time of this study recruited only HIV-positive individuals. Plasma CD4^+^ cell count and VL were readily available across studies, but these plasma markers are not comprehensive assessments of the full systemic impact of HIV. Future efforts are needed to pool and harmonize additional immunologic, cerebrovascular, metabolic, and inflammatory markers associated with infection. Identifying clinical factors that can be uniformly collected or interpreted across international studies remains challenging. For example, differences in treatment regimens with varying CNS penetration effectiveness scores, duration of treatment, and standards of adherence that qualified a participant categorically as taking cART at the time of MRI may vary from study to study. Despite such variations, this study identified consistent and robust brain volume associations with HIV VL and immunosuppression in a large and diverse study sample of HIV-positive individuals from around the world.

## Conclusions

This analysis demonstrates the feasibility and utility of a global collaborative initiative to understand the neurologic signatures of HIV infection. We invite other HIV researchers to join the ENIGMA-HIV consortium. With a greater collaborative effort, we will be able to assess factors that may modulate neurologic outcomes, including cART treatment regimens, comorbidities, coinfections, substance use, socioeconomic factors, and demographic factors, as well as the functional implications of such structural brain differences, in well-powered analyses. Understanding the neurobiological changes that may contribute to neuropsychiatric and cognitive outcomes in HIV-positive individuals is critical for identifying individuals at risk for neurologic symptoms, driving novel treatments that may protect the CNS, and monitoring treatment response.

## Notes

Supplement. **eMethods and eResults.** Supplementary Methods and Results **eTable 1.** Study Inclusion and Exclusion Criteria, by Site **eTable 2.** Study T1-Weighted MRI Acquisition Parameters, by Site **eTable 3.** Data Quality Assurance and Exclusion **eTable 4.** Detectable Viral Load Thresholds, by Site **eTable 5.** Age Feasibility Study Mega-Analysis Results **eTable 6.** HIV Subcortical Volume Comparisons with Healthy Lifespan Centiles **eTable 7.** Lateralized Associations with CD4+ Cell Counts and Detectable Viral Load **eTable 8.** Dichotomized CD4+ Cell Count Threshold (200/μL) Analysis Results **eTable 9.** Harmonized Viral Load Threshold (400 copies/mL) Analysis Results **eFigure 1.** Age Association Forest Plots: Putamen, Globus Pallidus, Hippocampus, and Thalamus **eFigure 2.** Age Association Forest Plots: Accumbens, Amygdala, Caudate, and Ventricles **eFigure 3.** Histogram of HIV-positive Subcortical Volumes with Respect to Healthy Lifespan Centile Values **eFigure 4.** Histogram of HIV-positive Subcortical Volumes with Respect to Healthy Lifespan Centile Values, by cART Status **eFigure 5.** Histogram of HIV-positive Subcortical Volumes with Respect to Healthy Lifespan Centile Values, by CD4+ Cell Count (> 200/μL) **eFigure 6.** Histogram of HIV-positive Subcortical Volumes with Respect to Healthy Lifespan Centile Values, by Viral Load Detectability **eFigure 7.** CD4+ Cell Count Association Forest Plots: Putamen, Globus Pallidus, Hippocampus, and Thalamus **eFigure 8.** CD4+ Cell Count Association Forest Plots: Accumbens, Amygdala, Caudate, and Ventricles **eFigure 9.** CD4+ Cell Count and Detectable Viral Load Effect Sizes in Males Compared to Females **eReferences**
