# Supplementary material for: Association of Immunosuppression and Viral Load With Subcortical Brain Volume in an International Sample of People Living With HIV
Source: JAMA Netw Open. 2021 Jan 15;4(1):e2031190. doi: 10.1001/jamanetworkopen.2020.31190 (PMC7811179; doi:10.1001/jamanetworkopen.2020.31190)
Supplement: Supplement. — eMethods and eResults. Supplementary Methods and Results eTable 1. Study Inclusion and Exclusion Criteria, by Site eTable 2. Study T1-Weighted MRI Acquisition Parameters, by Site eTable 3. Data Quality Assurance and Exclusion eTable 4. Detectable Viral Load Thresholds, by Site eTable 5. Age Feasibility Study Mega-Analysis Results eTable 6. HIV Subcortical Volume Comparisons with Healthy Lifespan Centiles eTable 7. Lateralized Associations with CD4+ Cell Counts and Detectable Viral Load eTable 8. Dichotomized CD4+ Cell Count Threshold (200/μL) Analysis Results eTable 9. Harmonized Viral Load Threshold (400 copies/mL) Analysis Results eFigure 1. Age Association Forest Plots: Putamen, Globus Pallidus, Hippocampus, and Thalamus eFigure 2. Age Association Forest Plots: Accumbens, Amygdala, Caudate, and Ventricles eFigure 3. Histogram of HIV-positive Subcortical Volumes with Respect to Healthy Lifespan Centile Values eFigure 4. Histogram of HIV-positive Subcortical Volumes with Respect to Healthy Lifespan Centile Values, by cART Status eFigure 5. Histogram of HIV-positive Subcortical Volumes with Respect to Healthy Lifespan Centile Values, by CD4+ Cell Count (> 200/μL) eFigure 6. Histogram of HIV-positive Subcortical Volumes with Respect to Healthy Lifespan Centile Values, by Viral Load Detectability eFigure 7. CD4+ Cell Count Association Forest Plots: Putamen, Globus Pallidus, Hippocampus, and Thalamus eFigure 8. CD4+ Cell Count Association Forest Plots: Accumbens, Amygdala, Caudate, and Ventricles eFigure 9. CD4+ Cell Count and Detectable Viral Load Effect Sizes in Males Compared to Females eReferences [file jamanetwopen-e2031190-s001.pdf]

Supplementary Online Content

Nir TM, Fouche JP, Ananworanich J, et al. Association of immunosuppression and viral load with subcortical brain volume in an international sample of people living with HIV. *JAMA Netw Open*. 2021;4(1):e2031190. doi:10.1001/jamanetworkopen.2020.31190

eMethods and eResults. Supplementary Methods and Results
eTable 1. Study Inclusion and Exclusion Criteria, by Site
eTable 2. Study T1-Weighted MRI Acquisition Parameters, by Site
eTable 3. Data Quality Assurance and Exclusion
eTable 4. Detectable Viral Load Thresholds, by Site
eTable 5. Age Feasibility Study Mega-Analysis Results
eTable 6. HIV Subcortical Volume Comparisons with Healthy Lifespan Centiles
eTable 7. Lateralized Associations with CD4+ Cell Counts and Detectable Viral Load
eTable 8. Dichotomized CD4+ Cell Count Threshold (200/μL) Analysis Results
eTable 9. Harmonized Viral Load Threshold (400 copies/mL) Analysis Results
eFigure 1. Age Association Forest Plots: Putamen, Globus Pallidus, Hippocampus, and Thalamus
eFigure 2. Age Association Forest Plots: Accumbens, Amygdala, Caudate, and Ventricles
eFigure 3. Histogram of HIV-positive Subcortical Volumes with Respect to Healthy Lifespan Centile Values
eFigure 4. Histogram of HIV-positive Subcortical Volumes with Respect to Healthy Lifespan Centile Values, by cART Status
eFigure 5. Histogram of HIV-positive Subcortical Volumes with Respect to Healthy Lifespan Centile Values, by CD4+ Cell Count (> 200/μL)
eFigure 6. Histogram of HIV-positive Subcortical Volumes with Respect to Healthy Lifespan Centile Values, by Viral Load Detectability
eFigure 7. CD4+ Cell Count Association Forest Plots: Putamen, Globus Pallidus, Hippocampus, and Thalamus
eFigure 8. CD4+ Cell Count Association Forest Plots: Accumbens, Amygdala, Caudate, and Ventricles
eFigure 9. CD4+ Cell Count and Detectable Viral Load Effect Sizes in Males Compared to Females
eReferences

This supplementary material has been provided by the authors to give readers additional information about their work.

© 2021 T. M. Nir et al. *JAMA Network Open.*

eMethods and eResults

Data Quality Assurance and Exclusion

For each ROI extracted with FreeSurfer, histograms were created from each site's data to investigate the normality of the data distribution, and statistical outliers were identified. If the mean of an individual's subcortical volume was more than 3 standard deviations from the mean for the site, it was flagged for a more extensive quality control and possible removal from the analysis. Participants with an ROI volume greater than five standard deviations from the mean across sites were removed. The initial number of participants from each study with MRI, and the number of participants excluded are found in **eTable 3.** Exclusion criteria at this stage included 1) missing clinical or demographic data, 2) any MRI artifacts, gross anatomical abnormalities, or regional volumes failing quality assurance, or 3) an ROI volume greater than five standard deviations from the mean across sites. An additional four transgender individuals across two sites were excluded.

Reporting of Effect Sizes

Effect sizes for dichotomous variables were estimated by converting *t*-values from random effects multiple linear regressions to *d* statistics:

$$d = \frac{t(n_1 + n_2)}{\sqrt{n_1 n_2} * \sqrt{df}}$$

where n_1 and n_2 are the sample size for each group. For continuous variables, effect sizes were estimated by converting *t*-values to partial correlation coefficients:

$$r = \frac{t}{\sqrt{t^2 + df}}$$

For both equations, df is the degrees of freedom as outputted by the 'nlme' package in R (version 3.2.3).

Age Feasibility Study Mega-Analysis

To ensure sufficient power to detect associations between brain measures and variables of interest from data pooled across studies, a preliminary analysis tested for associations between age and eight brain volumes. Random effects multivariable linear regressions were performed. Three fixed-effects covariates were included in the model: sex, the interaction between age and sex, and estimated total intracranial volume to adjust for variability in head size. To account for scanner effects, the data collection site was used as the random-effects grouping variable; there were 19 sites in all. Statistical analyses were conducted with the 'nlme' package in R (version 3.2.3). Effect sizes were estimated using the *r*-value (partial correlation coefficients) after accounting for all covariates. Significance was determined using the Bonferroni correction threshold ($p \leq 0.0063$).

Across the entire sample (N=1,203), older age was associated with smaller volumes of all subcortical structures and larger ventricular volumes (*r*-value range: 0.13-0.30), consistent with studies in seronegative adults[1]. Sex was a significant predictor in models for associations between age and the thalamus, putamen, globus pallidus, hippocampus, and amygdala ($p \leq 0.0063$). Age effects in the subset of participants taking cART, males, and females mirrored that of the full group. Participants not taking cART showed significant associations between age and caudate, putamen, globus pallidus, nucleus accumbens, and ventricular volumes. No significant age by sex interaction was detected.

© 2021 T. M. Nir et al. *JAMA Network Open.*

HIV Subcortical Volume Comparisons with Healthy Lifespan Centiles

In a recent study from the ENIGMA lifespan working group, subcortical volume trajectories across the lifespan were derived from 18,605 healthy participants pooled from 88 international samples[2]. As there was a lack of site and scanner matched HIV-negative controls in our ENIGMA-HIV sample, we used the centile curves to extrapolate how subcortical volumes in HIV+ individuals compared to age-matched healthy individuals. Given the wide variability in population demographics and scanners from which the volumes were extracted and reported curves estimated, they may incorporate sufficient variability to serve as a surrogate for matched controls.

For each subcortical volume, we used the discrete centile values reported for every 10 years of age in Dima et al. supplementary Table S6 to estimate whether the distribution of volumes in our HIV+ sample are similar to that expected for an age-matched healthy population.

We did this under the assumptions that:

1. for each volume, at each age provided, the distribution of volumes across the population was Gaussian, and
2. for all ages between those provided, (e.g, any age between 40 and 50), the distribution could be estimated by linearly interpolating the mean and variance of the data from the two closest ages. *Note:* In Dima et al. Table S6, the ages for which centile volumes are reported span 20 to 90 years of age discretized at 10-year intervals**.**

We used the 50th centile (C50; 50th percentile) column as the mean of the distribution and estimated the standard deviation by assuming that the 75th centile (C75) is 0.674 standard deviations above the mean, as would be the case for a Gaussian distribution. Therefore, as $z = (x-\mu)/\sigma$, we calculated the standard deviation for each structure, at each age provided, using the formula:

$$\sigma_{age} = (C75 - C50)*0.674$$

As the centiles were calculated non-parametrically, the distribution was not exactly Gaussian; the standard deviation calculated from the 25th centile was (in nearly all cases) smaller than that calculated with the 75th centile. Assuming a wider distribution is a more conservative approach as it is harder to reject the null with greater variability or more noise; we therefore used the larger of the two estimates.

Next, for each age represented in our HIV+ sample, we estimated the distribution of each subcortical volume by linearly interpolating the volumes at the provided surrounding ages (e.g., 40 and 50 for someone 43 years of age). As each individual's volume is from a different distribution, we standardized the subject-level volumes by converting them to *z*-scores and percentile estimates: (1) We first determined the *z*-score associated with the observed volume of that subject with respect to that expected distribution; (2) Then, for each individual observation, we randomly sampled 10,000 instances from the expected distribution and determined how many times the observed volume was less than or equal to the randomly drawn volumes. This resulted in an approximate percentile for the individual.

The probability density function (*pdf*) for the set of 1,203 paricipants' *z*-scores for each volume was then compared to the *pdf* of 1,203 randomly sampled draws from the standard normal distribution. The Kullback–Leibler divergence was then calculated using the KLD function from the "LaplacesDemon" library available in R, and the intrinsic discrepancy was used as a measure of deviation between the two distributions. These KLD values are provided in **eTable 6**. To determine whether this deviation was greater than would be expected by chance, and derive a non-parametric *p*-value estimate, we drew 1,203 random values from the normal distribution and used the KLD intrinsic discrepancy measure to compare their *pdf* to that obtained from the original random sample to which we compared the *pdf* of the HIV+ distribution; we then repeated this 10,000 times to determine how many times the observed was less than the random sample. The number of

© 2021 T. M. Nir et al. *JAMA Network Open.*

times our observation was less extreme (or one, whichever was greater) was then divided by total number of permutations to derive a *p*-value for the divergence.

A second method for determining whether the observed volumes were likely to come from the distributions derived from healthy controls made use of the percentiles derived as described above. If the set of HIV+ observations was derived from the healthy distribution, the percentiles are likely to be uniformly distributed. Therefore, we binned a uniform distribution [0,1] into 100 bins and performed a chi-squared goodness of fit test to determine whether the observed percentiles were approximately uniformly distributed. The chi-squared test was performed using the "chisq.test" function in R, with 10,000 simulated values. These *p*-values are also provided in **eTable 6**.

We note, all volumes for our HIV+ population appear to be from distributions that are not those described using healthy control data from multiple international imaging centers. Therefore, we anticipate significant case/control differences are likely across all structures, although the lack of HIV-negative controls from all sites prevented us from testing this directly.

For approximate visualization of distributions, we tabulated the number of HIV+ individuals in a given age range whose subcortical volumes fell between each pair of healthy centile values (e.g., for the hippocampus, how many HIV+ individuals ≤ 25 years of age and > 35 years fell between the C10 (10th percentile) and C25 (25th percentile) hippocampal volume values for age 30). The resulting histograms are shown in **eFigures 3-6**.

Validation Analyses

Two sets of validation analyses were performed: 1) dichotomizing CD4+ cell count based on the AIDS-defining threshold of 200/µL; and 2) defining a common dVL threshold across sites (400 copies/mL, the highest detection limit for any site). As in the primary analyses, multivariable random effects linear regressions were performed to evaluate associations between regional brain volumes and binary variables indicating 1) a CD4+ ≤ 200/µL (1) or CD4+ > 200/µL (0) and 2) a detectable (1) or undetectable (0) VL.

An AIDS-defining immunosuppression status (CD4+ ≤ 200/µL) was associated with smaller putamen volumes in participants not taking cART ($d = 0.41$, $p = 0.0007$; **eTable 7**). A dVL > 400 copies/mL was associated with larger ventricles in the full group ($d = 0.20$, $p = 0.0022$), participants taking cART ($d = 0.33$, $p = 0.0011$), and males ($d = 0.31$; $p = 0.0002$) (**eTable 8**). In both participants taking cART and males, a dVL was associated with smaller hippocampal volumes (taking cART: $d = -0.28$, $p = 0.0055$; Male: $d = -0.26$; $p = 0.0023$). Male participants with a dVL additionally had significantly smaller thalamic volumes ($d = -0.23$, $p = 0.0062$).

© 2021 T. M. Nir et al. *JAMA Network Open.*

eTable 1. Study Inclusion and Exclusion Criteria, by Site. Inclusion and exclusion criteria for study participants.

Site name	Inclusion Criteria	Exclusion Criteria	Reference
**HIVNC Consortium (7 Sites), United States**	HIV positive; Age > 18 years; On stable cART ≥ 12 weeks; Nadir CD4 < 200 cells/μL	Major psychiatric illness; Confounding neurological disorders; Brain infection other than HIV; Hepatic dysfunction; Diabetes mellitus; Active substance abuse or related medical complications within 6 months of study	Nir et al. 2019[3]
**University of Hawaii, United States (Chang/ Ernst)**	HIV positive; Age > 18; On stable cART > 6 months or no cART; Nadir CD4 ≤ 500 cells/μL.	Any illness known to alter brain structures; History of illicit drug or alcohol dependence by DSM-IV criteria; Positive urine toxicology screen; Head trauma with loss of consciousness > 30min; Severe abnormalities on screening laboratory tests that might confound brain imaging measures or function; Any contraindications for MRI.	Chang et al. 2011[4]
**University of Hawaii, United States (Shikuma)**	HIV positive; Age > 40 years; On stable cART ≥ 3 months	Uncontrolled major affective disorder; Active psychosis; Loss of consciousness > 5 min; Pregnant or breastfeeding; Any past or present confounding condition (e.g. CNS infection, TBI, stroke, substance abuse)	Kallianpur et al. 2016[5]
**University of California, San Francisco, United States**	HIV positive; Age > 60 years	Learning disabilities; Major psychiatric or neurological illness; Current or past brain infection; Major systemic illness or head injury	Clifford et al. 2017[6]
**Brown University, United States**	HIV positive; Age > 18 years<br><br>Note: Some HIV+ participants presented with Hepatitis C	History of head injury with loss of consciousness > 10 min; Neurological conditions including dementia unrelated to HIV, seizure disorder, stroke, and opportunistic infection of the brain; Severe psychiatric illness that may impact brain function (e.g., schizophrenia); Diagnosis of alcohol or substance abuse or dependence based within 6 months prior to neuroimaging	Gongvatana et al. 2014[7]
**University of California, Los Angeles, United States (Hinkin)**	HIV positive; Age > 18 years	Neurological, psychiatric, and medical confounds (e.g., history of seizure disorder or other neurologic disorder; history of concussion or TBI; history of Axis I psychiatric disorder or current substance use disorder; current prescription for psychotropic medication, except for anxiolytics and antidepressants; current substance dependence or methamphetamine use); Comorbid CNS infection (e.g. Hepatitis C); HIV-associated CNS opportunistic infection (e.g. toxoplasmosis) or neoplasm; MRI contraindications	Kuhn et al. 2017[8]
**University of California, Los Angeles, United States (Thames)**	HIV positive; Age > 18 years; Chronic HIV infection (mean duration of 12 years); On stable cART ≥ 3 months; Clinically stable (as indicated by CD4+ cells/μL); Score ≥ 26 on Mini-Mental Status Exam	Current abuse of cocaine/amphetamines; Past stimulant abuse/dependence; Current/past diagnosis of psychotic spectrum disorder; CNS confounds (e.g., HIV-associated opportunistic infections or neuro-syphilis); Hepatitis C coinfection; Major head injury; MRI contraindication	Thames et al. 2018[9]
**University of New South Wales, Australia (Brew)**	HIV positive; Age > 18 years; On stable cART ≥ 12 months	Contraindication to MRI; Comorbid CNS infection, tumor or stroke	Changanti et al 2017[10]

© 2021 T. M. Nir et al. *JAMA Network Open.*

**University of New South Wales, Australia (Cysique)**	HIV positive; Age > 45 years; On stable cART ≥ 6 months; Nadir CD4 ≤ 350 cells/μL; Known HIV duration ≥ 5 years; No active opportunistic disease<br><br>Note: HIV+ participants all male	MRI contraindication; History of neurological disorders predating HIV diagnosis (e.g., epilepsy, traumatic brain injury, Parkinson's Disease, Multiple Sclerosis, Alzheimer's disease, or Vascular Dementia); Psychiatric disorders on the psychotic axis (e.g., schizophrenia); Current substance use disorders (within 12 months of study enrolment; recreational use of marijuana was not set as a criterion for exclusion because it would exclude a large number of HIV+ individuals); Participants not excluded on the basis of current depressive symptoms; Hepatitis C status was recorded from the participants' medical records and participants were included only if successfully treated and/or inactive (N=2)	Nichols et al. 2019[11]
**SEARCH 011 Consortium, Thailand**	HIV positive; Age > 18 years; cART-naïve; CD4 < 350 cells/μL or symptomatic HIV	Head injury; Current substance abuse; Acute concurrent illness; Neurologic or psychiatric conditions; Learning disabilities; Positive hepatitis C screen; Contraindications for MRI; Significant laboratory abnormalities (e.g. creatinine, ALT, hemoglobin)	Heaps et al. 2015[12]
**University of Cape Town, South Africa**	HIV positive; Age 18 to 50 years to avoid CNS complications associated with neurodevelopment and advanced age; at least 5 years of formal education; cART initiation within 3 months of enrollment; Xhosa as the primary language<br><br>Note: Predominantly female; 100% detectable viral load	Major psychiatric conditions (schizophrenia, bipolar disorder, post- traumatic stress disorder, etc.); Neurological disease that could affect brain integrity (e.g., multiple sclerosis); CDC stage A; Opportunistic infections of the CNS (e.g., cytomegalovirus encephalitis, cryptococcal meningitis, toxo- plasma encephalitis); Lifetime history of head injury resulting in loss of consciousness >30 min; Current substance use disorder as determined by the Mini- International Neuropsychiatric Interview Plus	Paul et al. 2017[13]
**Nice University, France**	HIV positive; age > 18 years	CNS opportunistic infections; Change in psychotropic therapy < 3 weeks; Any neurological history	Vassallo et al. 2015[14]
**University of Novi Sad, Serbia**	HIV positive; Age > 18 yrs; On stable cART ≥ 12 months; Nadir CD4+ ≤ 250 cells/μL	A history of neurological disorders predating HIV (e.g., epilepsy, brain tumor, TBI, Parkinson's Disease, Multiple Sclerosis, Alzheimer's disease, or Vascular Dementia); Psychiatric disorders on the psychotic axis (e.g., schizophrenia); Cardiovascular diseases (hypertension, chronic occlusive carotid disease, ischemic vascular dementia); History of or current substance use disorders; Hepatitis C (HCV) or Hepatitis B positive	Boban et al. 2017[15]

© 2021 T. M. Nir et al. *JAMA Network Open.*

eTable 2. Study T1-Weighted MRI Acquisition Parameters, by Site.

Site	Scanner	Acquisition Parameters
**HIVNC Consortium, United States**	1.5T GE Signa, Siemens Symphony/Sonata	GE SPGR; TR=2-23 ms; TE=3-9 ms; flip angle=30°; matrix size = 256 × 128; voxel size=1x1x1.2-1.5 mm Siemens: TR=20-24 ms; TE=10.1 ms; flip angle = 30°; matrix size = 256 × 192; voxel size=1x1x1.2-1.3 mm
**University of Hawaii, United States (Chang/ Ernst)**	3T Siemens TrioTim	MP-RAGE; TR=2200 ms; TE=4.48 ms; TI=1000 ms; FOV = 256mm; 160 slices; voxel size=1x1x1mm
**University of Hawaii, United States (Shikuma)**	3T Phillips Achieva	3D TFE; TR=6.9 ms; TE = 3.2 ms; flip angle = 8°; FOV = 256mm; voxel size=1x1x1.2 mm
**University of California, San Francisco, United States**	3T Siemens TrioTim	MP-RAGE; TR=2300 ms; TE=2.98 ms; flip angle=9°; FOV = 256mm; 160 slices; matrix = 240×256; voxel size=1x1x1 mm
**Brown University, United States**	3T Siemens TrioTim	MP-RAGE; TR=2250 ms; TE=3.06 ms; flip angle=9°; FOV = 220 mm; matrix = 256×256, slice thickness = 0.86 mm
**University of California, Los Angeles, United States (Hinken)**	3T Siemens TrioTim	MP-RAGE; TR=2200 ms; TE=2.2 ms; matrix size = 256 × 256; FOV 240 mm; 176 slices; slice thickness = 1 mm
**University of California, Los Angeles, United States (Thames)**	3T Siemens TrioTim	MP-RAGE; TR = 450.0 ms; TE = 10.0 ms; flip angle: 8°; FOV 256 mm; matrix =256x219; voxel size =1.0x0.94x0.94 mm
**University of New South Wales, Australia (Brew)**	3T Philips Achieva	3D TFE; TR=5.3 ms; TE=2.4ms; flip angle = 18°, 256x256 matrix; 180 slices; voxel size=1x1x1 mm
**University of New South Wales, Australia (Cysique)**	3T Philips Achieva	3D TFE; TR= 6.39ms; TE=2.9 ms; flip angle: 8°; FOV 256 mm; 190 slices, voxel size=1x1x1 mm
**SEARCH 011 Consortium, Thailand**	3T Siemens Allegra	MP-RAGE; TR=2400 ms; TE=2.38 ms; flip angle=8°; 162 slices; voxel size=1x1x1 mm
**University of Cape Town, South Africa**	3T Siemens Allegra	MP-RAGE; TR=2400 ms; TE=2.38 ms; TI=1000 ms; flip angle=8°; 162 slices; voxel size=1x1x1 mm
**Nice University, France**	1.5T GE Signa	SPGR; TR =12.4 ms, TE = 5.2 ms, flip angle = 18°; FOV = 240 mm; 256x256 matrix; voxel size=0.6x0.6x0.6 mm
**University of Novi Sad, Serbia**	3T Siemens TrioTim	MP-RAGE; TR = 2300 ms; TE=2.97 ms; FOV 256 mm; voxel size=1x1x1 mm

© 2021 T. M. Nir et al. *JAMA Network Open.*

eTable 3. Data Quality Assurance and Exclusion. We note the initial number of participants with MRI from each study and the number of participants excluded from the final analysis. The row sum of excluded individuals may exceed the total number excluded as some participants met multiple exclusion criteria (i.e, often if age was missing, so was sex).

Site	Original N	Final N	Missing Data				Failed Quality Assurance	
			Demographic		Clinical		MRI/ FreeSurfer QC	> 5 SD from Mean
			Age	Sex	CD4+	cART		
**HIVNC Consortium, United States**	236	218	2	2	7	--	6	3
**University of Hawaii, United States (Chang/Ernst)**	192	175	--	1*	6	1	8	1
**University of Hawaii, United States (Shikuma)**	53	53	--	--	--	--	--	
**University of California, San Francisco, United States**	55	50	2	--	3	--	--	--
**Brown University, United States**	101	79	1	1	--	--	21	--
**University of California, Los Angeles, United States (Hinken)**	13	12	--	--	1	--	--	--
**University of California, Los Angeles, United States (Thames)**	70	51	--	3*	6	--	10	--
**University of New South Wales, Australia (Brew)**	42	39	--	--	1	--	--	--
**University of New South Wales, Australia (Cysique)**	82	68	--	--	--	--	14	--
**SEARCH 011 Consortium, Thailand**	62	61	--	--	1	--	--	--
**University of Cape Town, South Africa**	183	181	--	--	--	--	2	--
**Nice University, France**	161	155	--	--	--	--	6	--
**University of Novi Sad, Serbia**	87	61	6	--	4	12	9	--

* Transgender individuals

© 2021 T. M. Nir et al. *JAMA Network Open.*

eTable 4. Detectable Viral Load Thresholds, by Site. Approximate detectable plasma RNA viral load threshold for each site. The highest site-specific assay detection threshold, 400 copies/mL, was used to define a common, harmonized detectable VL threshold across all collection sites.

Site	Detectable Viral Load Threshold (copies/mL)
**HIVNC Consortium (7 Sites), United States**	50-400
**Site 1: University of California, San Diego**	50
**Site 2: Harbor UCLA Medical Center**	75
**Site 3: Stanford University**	50
**Site 4: Colorado**	50
**Site 5: Pittsburgh**	50
**Site 6: Rochester University**	50
**Site 7: University of California, Los Angeles**	400
**University of Hawaii, United States (Chang/ Ernst)**	75
**University of Hawaii, United States (Shikuma)**	50
**University of California, San Francisco, United States**	50
**Brown University, United States**	75
**University of California, Los Angeles, United States (Hinken)**	48
**University of California, Los Angeles, United States (Thames)**	19
**University of New South Wales, Australia (Brew)**	20
**University of New South Wales, Australia (Cysique)**	50
**SEARCH 011 Study, Thailand**	40
**University of Cape Town, South Africa**	50
**Nice University, France**	40
**University of Novi Sad, Serbia**	50

© 2021 T. M. Nir et al. *JAMA Network Open.*

eTable 5. Age Feasibility Study Mega-Analysis Results. Partial correlation coefficients (*r*), unstandardized regression slopes (*b*-values reflecting change in volume (mm^3) for every year of age), standard errors (SE), and *p*-values from associations between age and regional brain volumes across all HIV+ participants, and by cART status and sex.

ROI	Total (n=1,203)				Taking cART (n=897)				Not Taking cART (n=306)				Male (n=880)				Female (n=323)			
	r	*b*	SE	*p*	*r*	*b*	SE	*p*	*r*	*b*	SE	*p*	*r*	*b*	SE	*p*	*r*	*b*	SE	*p*
Thalamus	-0.25	-26.20	2.92	1.11E-18**	-0.31	-33.69	3.55	2.03E-20**	-0.12	-13.62	6.43	0.035*	-0.35	-29.66	2.68	1.02E-26**	-0.24	-22.32	5.22	2.53E-5**
Caudate	-0.13	-7.13	1.59	8.47E-6**	-0.10	-6.04	2.02	0.0029**	-0.21	-12.29	3.39	3.40E-4**	-0.16	-7.26	1.48	1.21E-6**	-0.16	-8.29	2.91	0.0046**
Putamen	-0.30	-24.30	2.26	8.21E-26**	-0.28	-25.58	2.94	1.49E-17**	-0.27	-21.37	4.41	2.06E-6**	-0.36	-24.86	2.20	8.23E-28**	-0.37	-26.38	3.76	1.41E-11**
Globus Pallidus	-0.21	-6.07	0.81	1.65E-13**	-0.20	-6.30	1.07	5.37E-9**	-0.22	-6.23	1.61	1.29E-4**	-0.24	-5.61	0.79	2.25E-12**	-0.30	-7.59	1.37	6.21E-8**
Hippocampus	-0.17	-9.12	1.55	5.02E-9**	-0.16	-9.41	1.96	1.77E-6**	-0.13	-7.27	3.32	0.029*	-0.24	-10.57	1.47	1.20E-12**	-0.16	-7.67	2.71	0.005**
Amygdala	-0.15	-3.77	0.72	1.62E-7**	-0.14	-3.75	0.91	4.40E-5**	-0.11	-2.76	1.43	0.054*	-0.21	-4.21	0.68	9.56E-10**	-0.18	-3.95	1.26	0.0019**
Accumbens	-0.26	-3.33	0.35	2.49E-20**	-0.24	-3.29	0.45	9.39E-13**	-0.24	-3.17	0.74	2.51E-5**	-0.29	-3.03	0.35	9.34E-18**	-0.34	-3.92	0.61	5.70E-10**
Lateral Ventricles	0.25	350.29	39.43	2.35E-18**	0.23	378.63	53.16	2.22E-12**	0.16	211.92	74.93	0.005**	0.33	397.61	39.39	1.02E-22**	0.23	238.27	56.98	3.78E-5**

**Significant at Bonferroni corrected threshold for tests in 8 regions of interest, $p \leq 0.0063$

*Suggestive at $p \leq 0.05$

© 2021 T. M. Nir et al. *JAMA Network Open.*

eTable 6. HIV Subcortical Volume Comparisons with Healthy Lifespan Centiles. Results from the Kullback–Leibler divergence, corresponding permutations, and chi-squared goodness of fit tests used to determine whether the observed volumes in the full HIV+ group, and subgroups stratified by cART status, CD4+ cell count, and viral load (VL), were likely to come from the distributions derived from healthy controls.

ROI	All HIV+			Taking cART			Not Taking cART			CD4+ ≤ 200/μL			CD4+ > 200/μL			dVL			uVL		
	KLD	KLD *P*-Value	χ2 *P*-Value	KLD	KLD *P*-Value	χ2 *P*-Value	KLD	KLD *P*-Value	χ2 *P*-Value	KLD	KLD *P*-Value	χ2 *P*-Value	KLD	KLD *P*-Value	χ2 *P*-Value	KLD	KLD *P*-Value	χ2 *P*-Value	KLD	KLD *P*-Value	χ2 *P*-Value
**L Thalamus**	0.41	0.0001	0.0001	0.38	0.0001	0.0001	0.42	0.0001	0.0001	0.41	0.0001	0.0001	0.37	0.0001	0.0001	0.41	0.0001	0.0001	0.39	0.0001	0.0001
**R Thalamus**	0.41	0.0001	0.0001	0.41	0.0001	0.0001	0.44	0.0001	0.0001	0.42	0.0001	0.0001	0.44	0.0001	0.0001	0.39	0.0001	0.0001	0.41	0.0001	0.0001
**L Hippocampus**	0.34	0.0001	0.0001	0.39	0.0001	0.0001	0.34	0.0001	0.0001	0.35	0.0001	0.0001	0.37	0.0001	0.0001	0.40	0.0001	0.0001	0.35	0.0001	0.0001
**R Hippocampus**	0.37	0.0001	0.0001	0.39	0.0001	0.0001	0.40	0.0001	0.0001	0.37	0.0001	0.0001	0.35	0.0001	0.0001	0.35	0.0001	0.0001	0.37	0.0001	0.0001
**L Ventricle**	0.29	0.0001	0.0001	0.34	0.0001	0.0001	0.29	0.0001	0.0001	0.31	0.0001	0.0001	0.33	0.0001	0.0001	0.30	0.0001	0.0001	0.32	0.0001	0.0001
**R Ventricle**	0.34	0.0001	0.0001	0.32	0.0001	0.0001	0.30	0.0009	0.0001	0.32	0.0001	0.0001	0.29	0.0001	0.0001	0.30	0.0001	0.0001	0.27	0.0002	0.0001
**L Amygdala**	0.37	0.0001	0.0001	0.43	0.0001	0.0001	0.42	0.0001	0.0001	0.39	0.0001	0.0001	0.37	0.0001	0.0001	0.43	0.0001	0.0001	0.38	0.0001	0.0001
**R Amygdala**	0.43	0.0001	0.0001	0.44	0.0001	0.0001	0.39	0.0001	0.0001	0.44	0.0001	0.0001	0.42	0.0001	0.0001	0.42	0.0001	0.0001	0.44	0.0001	0.0001
**L Caudate**	0.30	0.0001	0.0001	0.29	0.0001	0.0001	0.28	0.0001	0.0001	0.31	0.0001	0.0001	0.32	0.0001	0.0001	0.33	0.0001	0.0001	0.28	0.0001	0.0002
**R Caudate**	0.36	0.0001	0.0001	0.33	0.0001	0.0001	0.33	0.0001	0.0001	0.32	0.0001	0.0658	0.33	0.0001	0.0001	0.34	0.0001	0.0001	0.28	0.0004	0.0001
**L Globus Pallidus**	0.39	0.0001	0.0001	0.40	0.0001	0.0001	0.37	0.0001	0.0001	0.41	0.0001	0.0001	0.39	0.0001	0.0001	0.41	0.0001	0.0001	0.40	0.0001	0.0001
**R Globus Pallidus**	0.39	0.0001	0.0001	0.37	0.0001	0.0001	0.40	0.0001	0.0001	0.37	0.0001	0.0001	0.42	0.0001	0.0001	0.36	0.0001	0.0001	0.39	0.0001	0.0001
**L Putamen**	0.40	0.0001	0.0001	0.37	0.0001	0.0001	0.38	0.0001	0.0001	0.43	0.0001	0.0001	0.38	0.0001	0.0001	0.36	0.0001	0.0001	0.34	0.0001	0.0001
**R Putamen**	0.38	0.0001	0.0001	0.38	0.0001	0.0001	0.38	0.0001	0.0001	0.42	0.0001	0.0001	0.42	0.0001	0.0001	0.36	0.0001	0.0001	0.38	0.0001	0.0001
**L Accumbens**	0.36	0.0001	0.0001	0.36	0.0001	0.0001	0.36	0.0001	0.0002	0.35	0.0001	0.0081	0.35	0.0001	0.0001	0.36	0.0001	0.0002	0.36	0.0001	0.0001
**R Accumbens**	0.43	0.0001	0.0001	0.40	0.0001	0.0001	0.42	0.0001	0.0001	0.37	0.0001	0.0001	0.42	0.0001	0.0001	0.40	0.0001	0.0001	0.38	0.0001	0.0001

© 2021 T. M. Nir et al. *JAMA Network Open.*

eTable 7. Lateralized Associations with CD4+ Cell Counts and Detectable Viral Load. Associations between left and right subcortical volumes and CD4+ cell count or detectable viral load (dVL) at the time of scan for regions that showed significant associations when averaged bilaterally in primary analyses across HIV+ participants.

**ROI**	**CD4+ (n=1,203)**								**dVL (n=1,161)**					
	**Left**				**Right**				**Left**			**Right**		
	r	*b*	*SE*	*p*	*r*	*b*	*SE*	*p*	*d*	*SE*	*p*	*d*	*SE*	*p*
**Thalamus**	0.093	31.18	9.73	0.0014**	0.10	32.84	9.14	0.0003**	--	--	--	--	--	--
**Hippocampus**	0.11	18.23	5.02	0.0003**	0.090	15.23	4.90	0.0019**	-0.16	0.060	0.0066*	-0.16	0.060	0.0074*
**Lateral Ventricles**	-0.091	-204.52	65.49	0.0018**	-0.090	-188.92	60.43	0.0019**	--	--	--	--	--	--

**Significant at Bonferroni corrected threshold for tests in 8 regions of interest $p \leq 0.0063$
*Suggestive at $p \leq 0.05$

© 2021 T. M. Nir et al. *JAMA Network Open.*

eTable 8. Dichotomized CD4+ Cell Count Threshold Analysis Results. Effect sizes (*d*) for associations between regional brain volumes and an AIDS-defining immunosuppression status (CD4+ cell count ≤ 200/µL) across HIV+ individuals, and by cART status and sex.

ROI	Total (n=1,203)			Taking cART (n=897)			Not Taking cART (n=306)			Male (n=880)			Female (n=323)		
	d	**SE**	***p***	***d***	**SE**	***p***	***d***	**SE**	***p***	***d***	**SE**	***p***	***d***	**SE**	***p***
**Thalamus**	-0.065	0.073	0.38	0.014	0.10	0.89	-0.18	0.12	0.135	0.028	0.095	0.77	-0.19	0.12	0.12
**Caudate**	-0.057	0.073	0.44	-0.047	0.10	0.66	-0.081	0.12	0.49	-0.067	0.095	0.49	-0.012	0.12	0.93
**Putamen**	-0.18	0.073	0.017*	-0.057	0.10	0.59	**-0.41**	**0.12**	**0.0007****	-0.19	0.095	0.046*	-0.16	0.12	0.21
**Globus Pallidus**	-0.12	0.073	0.094	-0.052	0.10	0.62	-0.28	0.12	0.021*	-0.10	0.095	0.29	-0.13	0.12	0.30
**Hippocampus**	-0.16	0.073	0.034*	-0.087	0.10	0.41	-0.26	0.12	0.029*	-0.19	0.095	0.045*	-0.095	0.12	0.44
**Amygdala**	-0.13	0.073	0.076	-0.092	0.10	0.38	-0.22	0.12	0.070	-0.12	0.095	0.20	-0.15	0.12	0.22
**Accumbens**	-0.15	0.073	0.049*	-0.060	0.10	0.57	-0.29	0.12	0.016*	-0.063	0.095	0.51	-0.31	0.12	0.013*
**Lateral Ventricles**	0.098	0.073	0.19	0.084	0.10	0.42	0.14	0.12	0.24	0.095	0.095	0.32	0.16	0.12	0.20
**% with CD4+ Count < 200/µL**	19.1%			11.9%			40.2%			14.9 %			30.7%		

**Significant at Bonferroni corrected threshold for tests in 8 regions of interest, $p \leq 0.0063$
*Suggestive at $p \leq 0.05$

eTable 9. Harmonized Viral Load Threshold Analysis Results. Effect sizes (*d*) for associations between regional brain volumes and dVL > 400 copies/mL across HIV+ individuals, and by cART status and sex. We note that dVL associations in those off treatment included only a limited number of individuals with undetectable VL (n=24), and should be interpreted with caution.

ROI	Total (n=1,156)			Taking cART (n=884)			Not Taking cART (n=272)			Male (n=863)			Female (n=293)		
	d	**SE**	***p***	***d***	**SE**	***p***	***d***	**SE**	***p***	***d***	**SE**	***p***	***d***	**SE**	***p***
**Thalamus**	-0.15	0.063	0.016*	-0.26	0.10	0.012*	-0.11	0.21	0.60	**-0.23**	**0.083**	**0.0062****	0.063	0.12	0.61
**Caudate**	-0.017	0.063	0.79	0.020	0.10	0.84	-0.22	0.21	0.30	0.007	0.082	0.94	-0.088	0.12	0.48
**Putamen**	-0.021	0.063	0.74	-0.037	0.10	0.71	0.006	0.21	0.98	-0.049	0.082	0.56	0.025	0.12	0.84
**Globus Pallidus**	-0.041	0.063	0.52	0.006	0.10	0.95	-0.38	0.21	0.08	-0.019	0.082	0.82	-0.14	0.12	0.25
**Hippocampus**	-0.16	0.063	0.011*	**-0.28**	**0.10**	**0.0055****	-0.004	0.21	0.99	**-0.26**	**0.083**	**0.0023****	0.031	0.12	0.80
**Amygdala**	-0.079	0.063	0.22	-0.14	0.10	0.17	-0.003	0.21	0.99	-0.12	0.083	0.16	-0.006	0.12	0.96
**Accumbens**	-0.11	0.063	0.098	-0.19	0.10	0.060	0.037	0.21	0.87	-0.22	0.083	0.009*	0.18	0.12	0.15
**Lateral Ventricles**	**0.20**	**0.064**	**0.0022****	**0.33**	**0.10**	**0.0011****	0.024	0.21	0.91	**0.31**	**0.083**	**0.0002****	-0.078	0.12	0.53
**% with Viral Load > 400 copies/mL**	31.4%			13.0%			91.2%			21.8%			59.7%		

**Significant at Bonferroni corrected threshold for tests in 8 regions of interest, $p \leq 0.0063$
*Suggestive at $p \leq 0.05$

© 2021 T. M. Nir et al. *JAMA Network Open.*

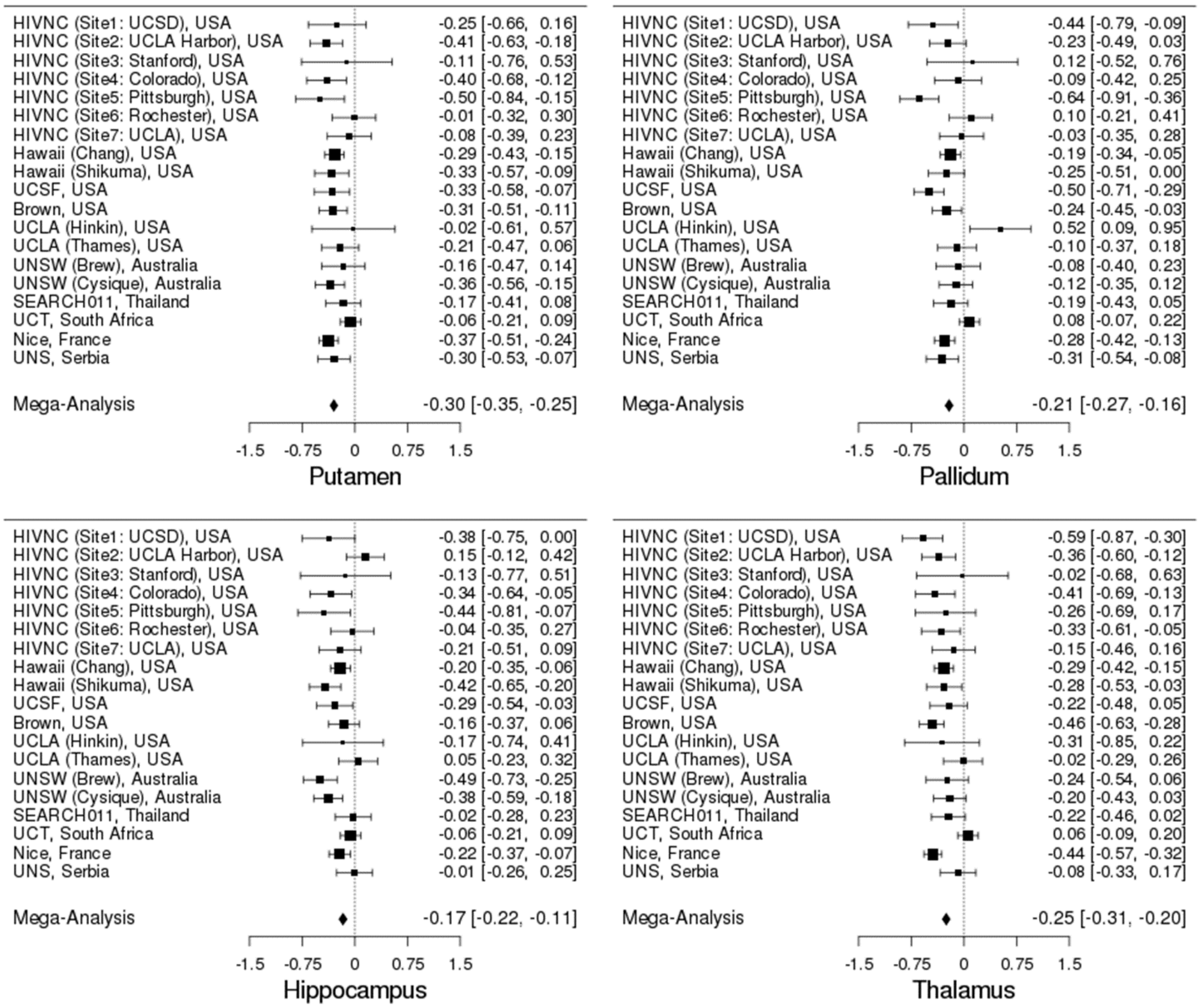

eFigure 1. Age Association Forest Plots: Putamen, Globus Pallidus, Hippocampus, and Thalamus. Forest plot of effect sizes (*r*-values and 95% confidence intervals) for associations between age and putamen, globus pallidus, hippocampal or thalamic volumes across all 19 sites.

© 2021 T. M. Nir et al. *JAMA Network Open.*

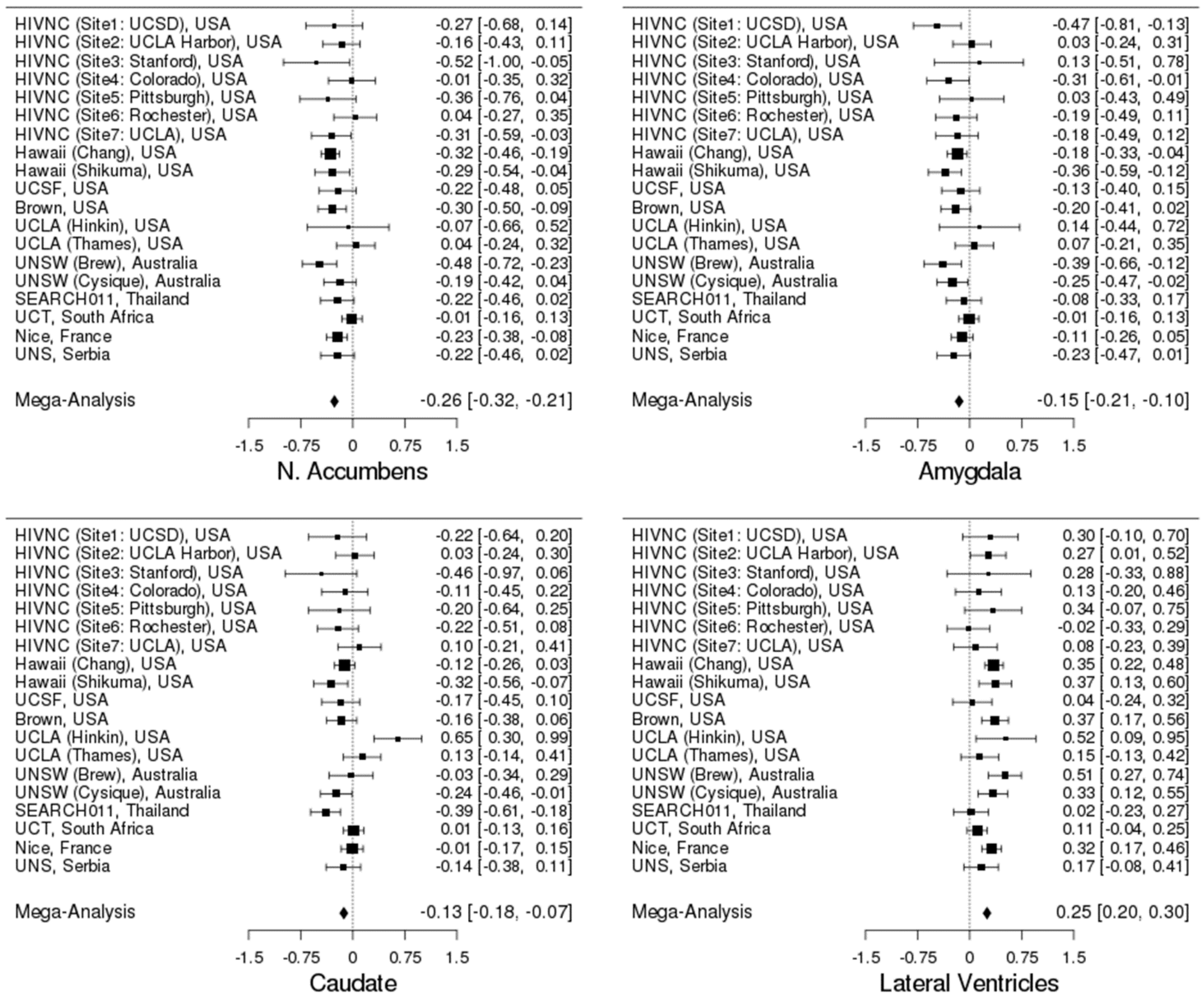

eFigure 2. Age Association Forest Plots: Accumbens, Amygdala, Caudate, and Ventricles. Forest plot of effect sizes (*r*-values and 95% confidence intervals) for associations between age and accumbens, amygdala, caudate or ventricular volumes across 19 sites.

© 2021 T. M. Nir et al. *JAMA Network Open.*

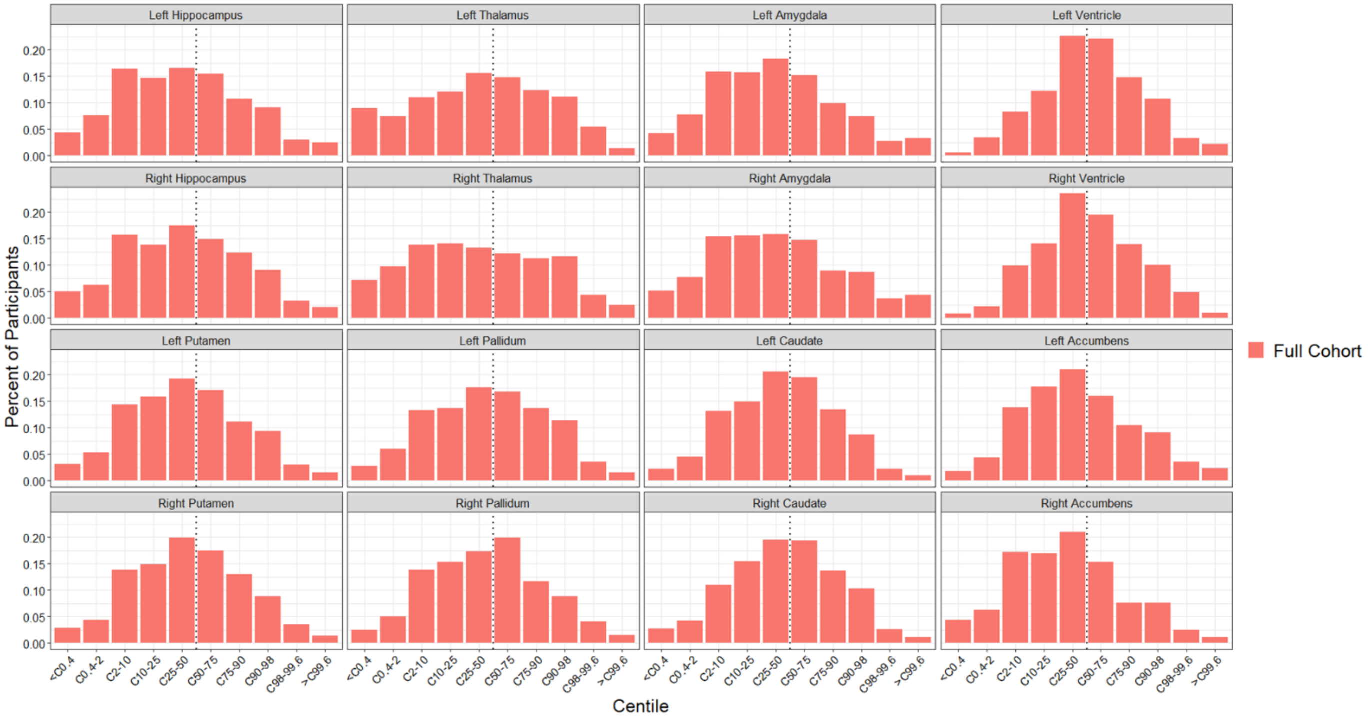

eFigure 3. Histogram of HIV-positive Subcortical Volumes with Respect to Healthy Lifespan Centile Values. Percent of HIV+ participants, across the full cohort, with subcortical volumes that fall into each healthy subcortical volume centile bin as defined in Table S6 of Dima et al. (2020). The dotted line marks the 50th percentile.

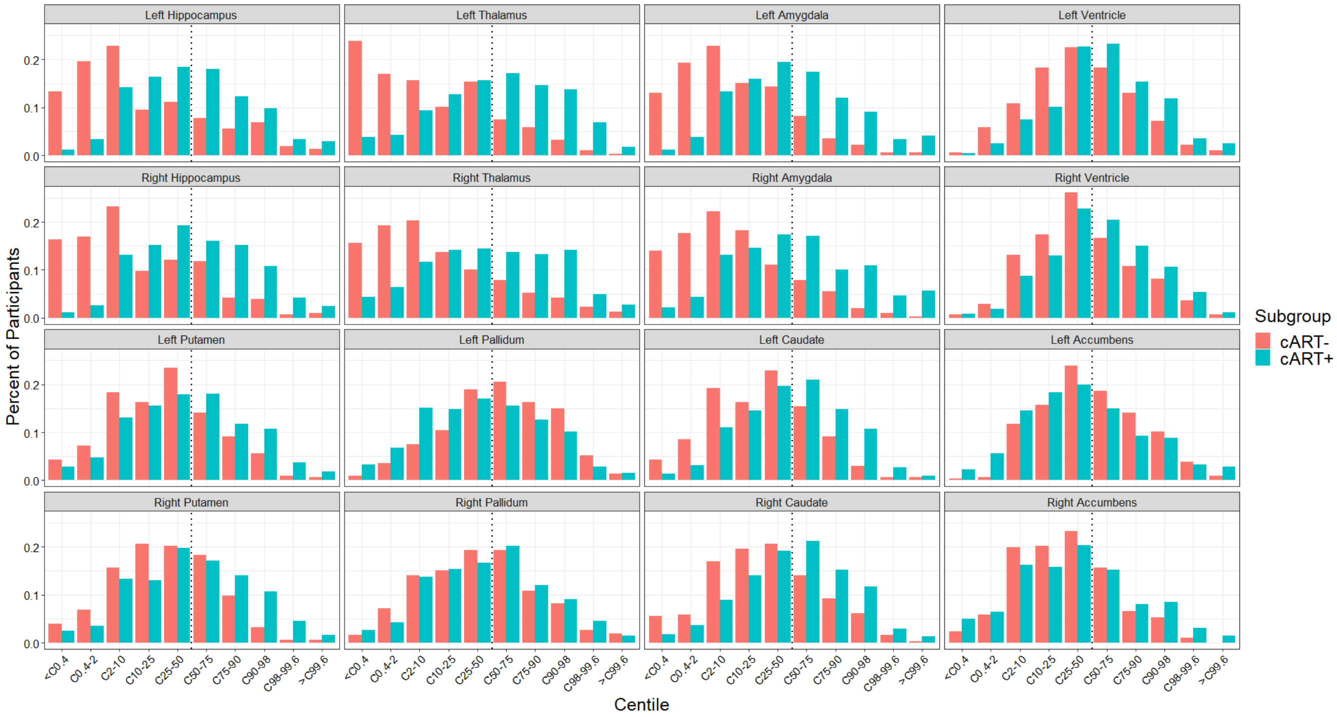

eFigure 4. Histogram of HIV-positive Subcortical Volumes with Respect to Healthy Lifespan Centile Values, by cART Status. Percent of HIV+ participants taking or not taking cART (cART+/cART-) with subcortical volumes that fall into each healthy subcortical volume centile bin as defined in Table S6 of Dima et al. (2020). The dotted line marks the 50th percentile.

© 2021 T. M. Nir et al. *JAMA Network Open.*

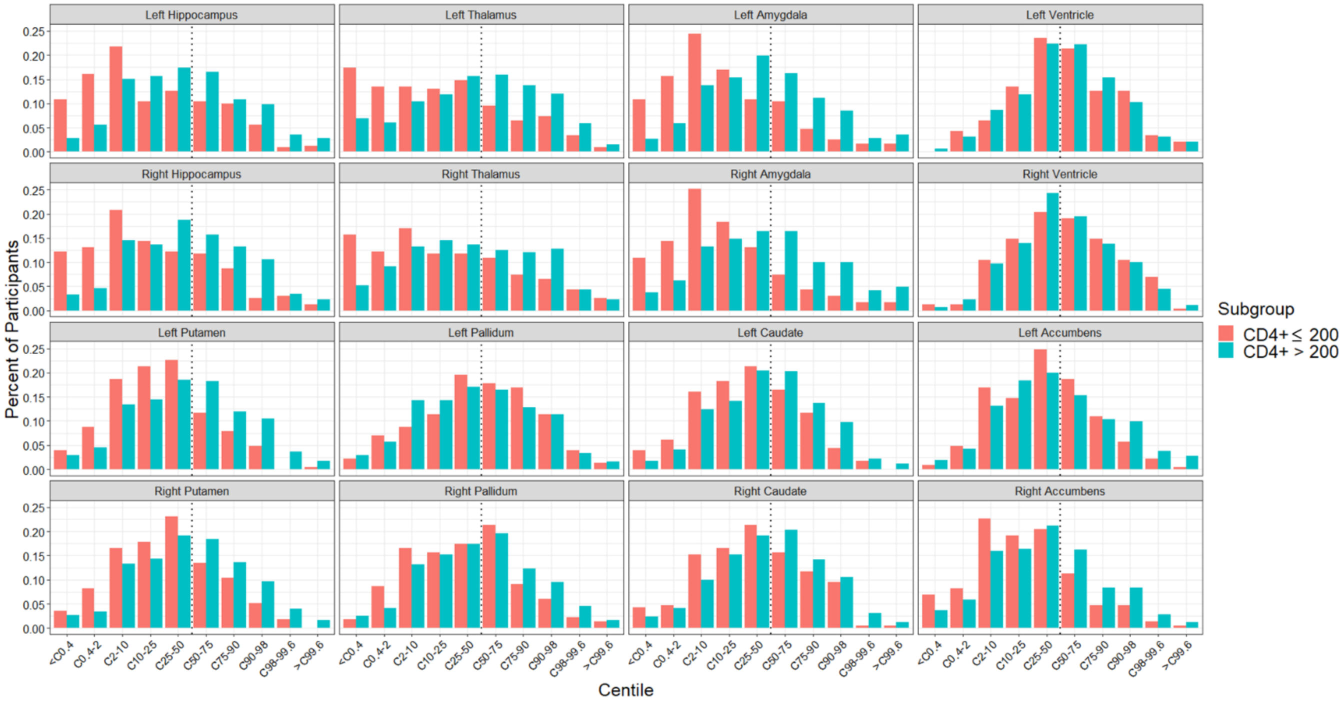

eFigure 5. Histogram of HIV-positive Subcortical Volumes with Respect to Healthy Lifespan Centile Values, by CD4+ Cell Count (> 200/µL). Percent of HIV+ participants with either CD4+ cell count ≤ 200/µL or > 200/µL with subcortical volumes that fall into each healthy subcortical volume centile bin as defined in Table S6 of Dima et al. (2020). The dotted line marks the 50th percentile.

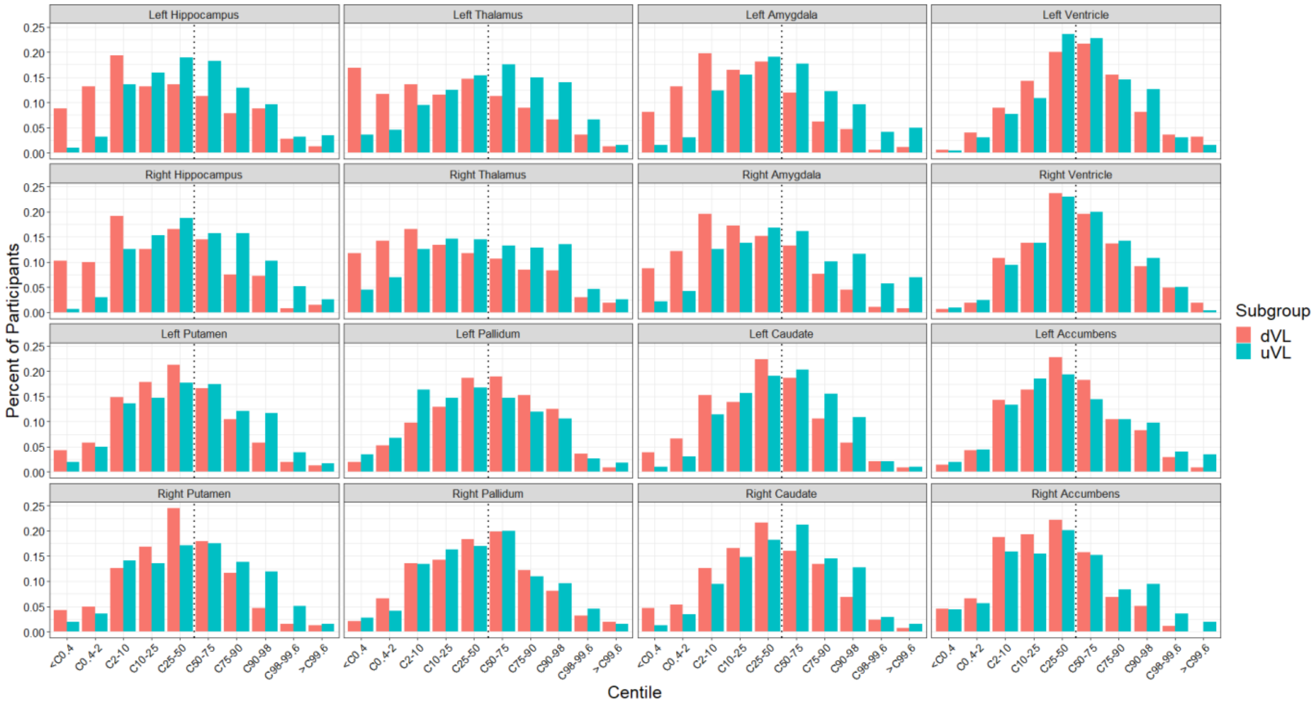

eFigure 6. Histogram of HIV-positive Subcortical Volumes with Respect to Healthy Lifespan Centile Values, by Viral Load Detectability. Percent of HIV+ participants with either detectable or undetectable viral loads (dVL or uVL, respectively) with subcortical volumes that fall into each healthy subcortical volume centile bin as defined in Table S6 of Dima et al. (2020). The dotted line marks the 50th percentile.

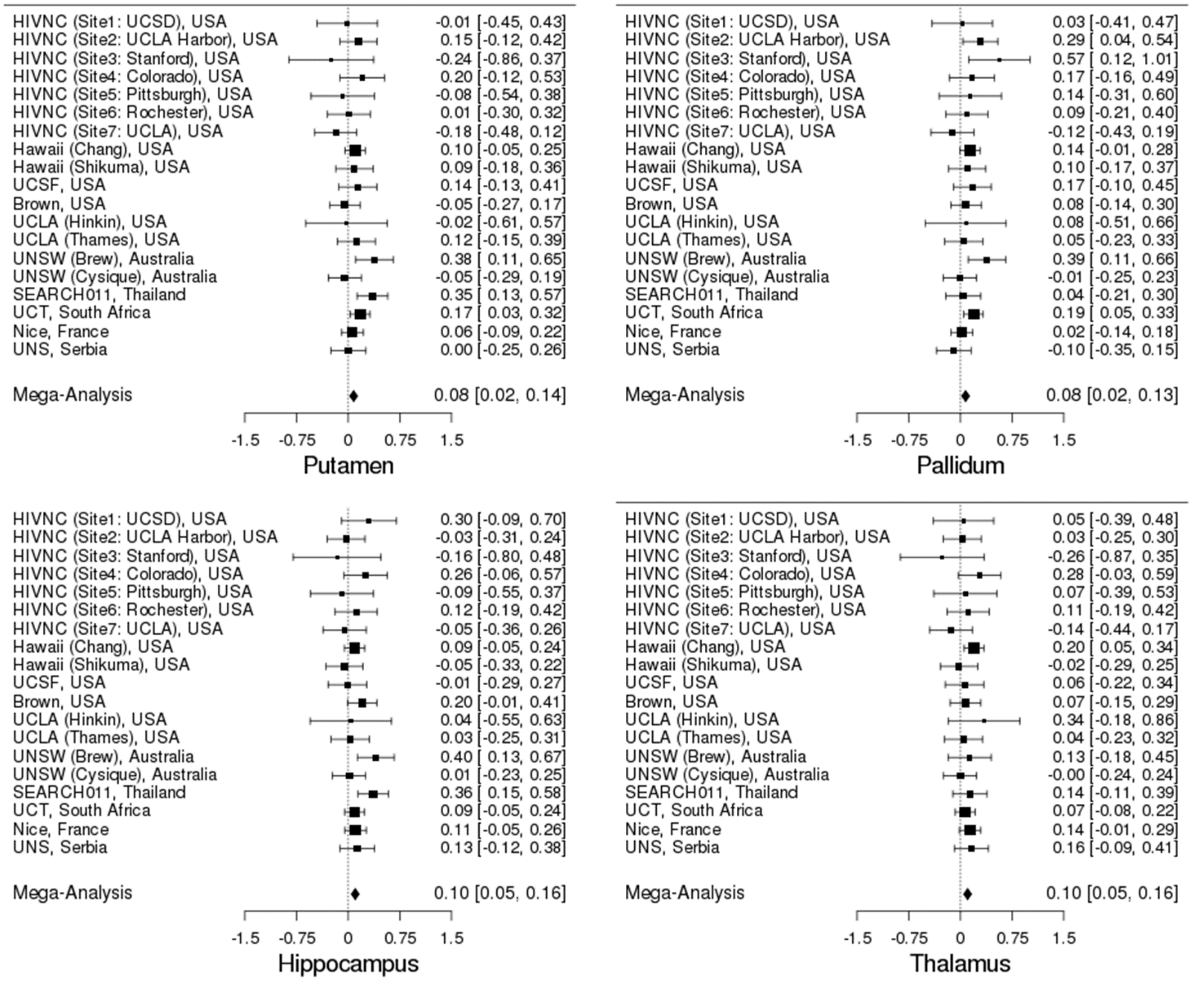

eFigure 7. CD4+ Cell Count Association Forest Plots: Putamen, Globus Pallidus, Hippocampus, and Thalamus. Forest plot of effect sizes (*r*-values and 95% confidence intervals) for associations between CD4+ cell count and putamen, globus pallidus, hippocampal or thalamic volumes across 19 sites.

© 2021 T. M. Nir et al. *JAMA Network Open.*

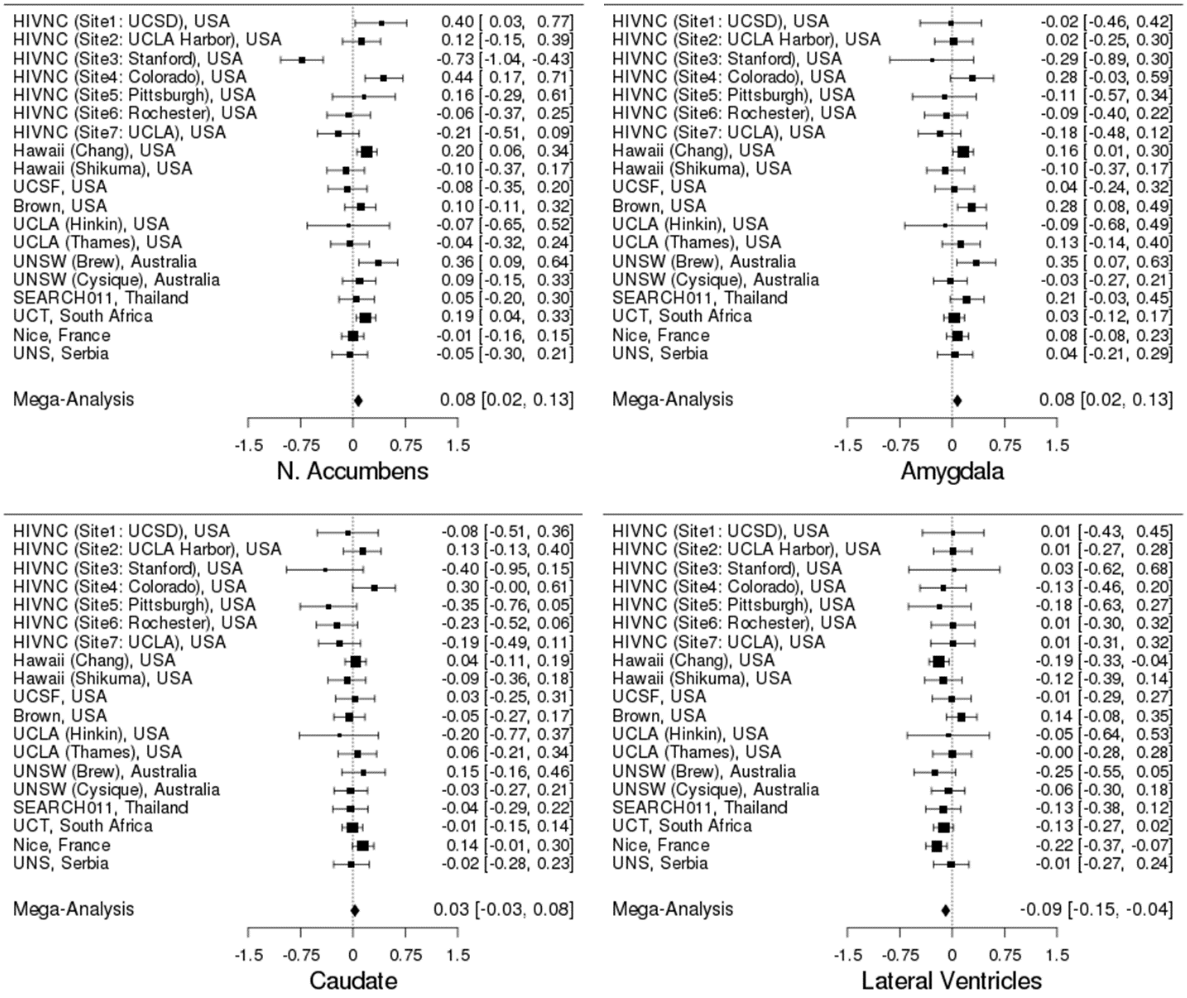

eFigure 8. CD4+ Cell Count Association Forest Plots: Accumbens, Amygdala, Caudate, and Ventricles. Forest plot of effect sizes (*r*-values and 95% confidence intervals) for associations between CD4+ cell count and accumbens, amygdala, caudate or ventricular volumes across 19 sites.

© 2021 T. M. Nir et al. *JAMA Network Open.*

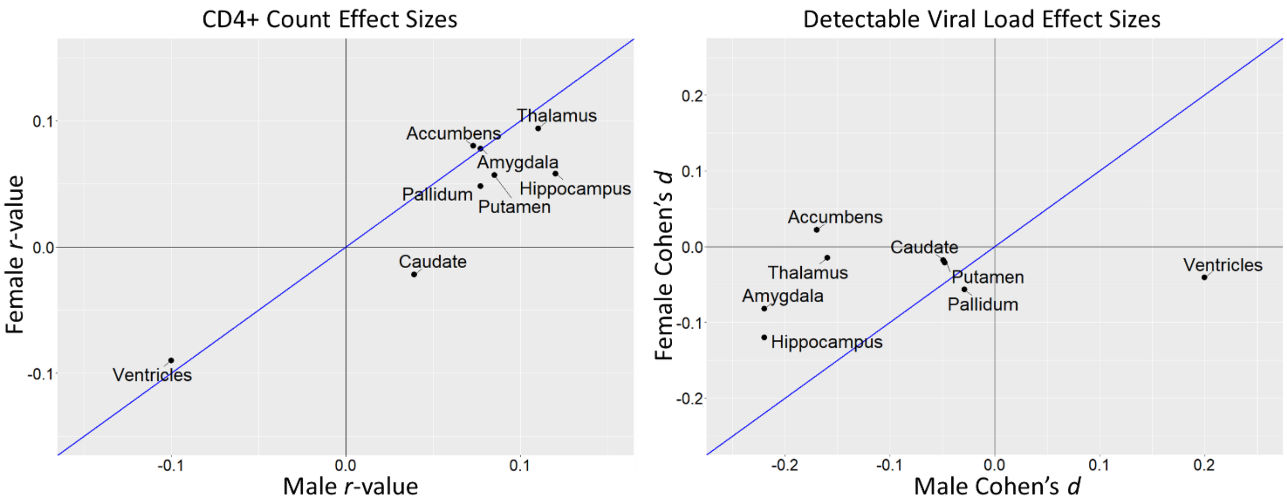

eFigure 9. CD4+ Cell Count and Detectable Viral Load Effect Sizes in Males Compared to Females. Effect sizes in males compared to females for each ROI were highly correlated (CD4+: Pearson's correlation r = 0.91, p =0.002; dVL: r = 0.22, p = 0.60).

eReferences

1. Potvin O, Mouiha A, Dieumegarde L, Duchesne S, Alzheimer's Disease Neuroimaging I. Normative data for subcortical regional volumes over the lifetime of the adult human brain. *Neuroimage.* 2016;137:9-20.
2. Dima D, Papachristou E, Modabbernia A, et al. Subcortical Volume Trajectories across the Lifespan: Data from 18,605 healthy individuals aged 3-90 years. *bioRxiv.* 2020:2020.2005.2005.079475.
3. Nir TM, Jahanshad N, Ching CRK, et al. Progressive brain atrophy in chronically infected and treated HIV+ individuals. *Journal of neurovirology.* 2019;25(3):342-353.
4. Chang L, Andres M, Sadino J, et al. Impact of apolipoprotein E epsilon4 and HIV on cognition and brain atrophy: antagonistic pleiotropy and premature brain aging. *Neuroimage.* 2011;58(4):1017-1027.
5. Kallianpur KJ, Sakoda M, Gangcuangco LM, et al. Frailty Characteristics in Chronic HIV Patients are Markers of White Matter Atrophy Independently of Age and Depressive Symptoms: A Pilot Study. *Open Med J.* 2016;3:138-152.
6. Clifford KM, Samboju V, Cobigo Y, et al. Progressive Brain Atrophy Despite Persistent Viral Suppression in HIV Patients Older Than 60 Years. *Journal of acquired immune deficiency syndromes (1999).* 2017;76(3):289-297.
7. Gongvatana A, Correia S, Dunsiger S, et al. Plasma cytokine levels are related to brain volumes in HIV-infected individuals. *J Neuroimmune Pharmacol.* 2014;9(5):740-750.
8. Kuhn T, Schonfeld D, Sayegh P, et al. The effects of HIV and aging on subcortical shape alterations: A 3D morphometric study. *Hum Brain Mapp.* 2017;38(2):1025-1037.
9. Thames AD, Kuhn TP, Mahmood Z, et al. Effects of social adversity and HIV on subcortical shape and neurocognitive function. *Brain Imaging Behav.* 2018;12(1):96-108.
10. Chaganti JR, Heinecke A, Gates TM, Moffat KJ, Brew BJ. Functional Connectivity in Virally Suppressed Patients with HIV-Associated Neurocognitive Disorder: A Resting-State Analysis. *AJNR American journal of neuroradiology.* 2017;38(8):1623-1629.
11. Nichols MJ, Gates TM, Soares JR, et al. Atrophic brain signatures of mild forms of neurocognitive impairment in virally suppressed HIV infection. *AIDS.* 2019;33(1):55-66.
12. Heaps JM, Sithinamsuwan P, Paul R, et al. Association between brain volumes and HAND in cART-naive HIV+ individuals from Thailand. *Journal of neurovirology.* 2015;21(2):105-112.
13. Paul RH, Phillips S, Hoare J, et al. Neuroimaging abnormalities in clade C HIV are independent of Tat genetic diversity. *Journal of neurovirology.* 2017;23(2):319-328.
14. Vassallo M, Durant J, Lebrun-Frenay C, et al. Virologically suppressed patients with asymptomatic and symptomatic HIV-associated neurocognitive disorders do not display the same pattern of immune activation. *HIV Med.* 2015;16(7):431-440.
15. Boban J, Kozic D, Turkulov V, et al. HIV-associated neurodegeneration and neuroimmunity: multivoxel MR spectroscopy study in drug-naive and treated patients. *Eur Radiol.* 2017;27(10):4218-4236.

© 2021 T. M. Nir et al. *JAMA Network Open.*
